# Polyphasic taxonomic characterization of *Brachybacterium netajii* sp. nov., a metabolically versatile bacterium isolated from the river Ganges, India

**DOI:** 10.1038/s41598-026-56775-0

**Published:** 2026-06-16

**Authors:** Sk Aftabul Alam, Debabrata Karmakar, Biswajit Khan, Rajkumar Mandal, Satyabrata Bhattacharya, Ijaz Ahmed, Fumito Maruyama, Pradipta Saha

**Affiliations:** 1 Department of Botany, Netaji Mahavidyalaya, Arambagh, Hooghly, WB India; 2 Department of Microbiology, The University of Burdwan, Golapbag, Burdwan, West Bengal 713104 India; 3 Department of Pharmaceutical Engineering & Technology, Indian Institute of Technology, BHU, Varanasi, India; 4 The IDEC Institute, Hiroshima University, Higashi-Hiroshima, Japan

**Keywords:** Biotechnology, Computational biology and bioinformatics, Genetics, Microbiology, Molecular biology

## Abstract

A comprehensive polyphasic taxonomic strategy was applied to the systematic characterization of strain DNPG3^T^, which was isolated from the river Ganges, Hooghly, West Bengal, India. The Gram-positive, halotolerant, heavy-metal-tolerant strain exhibited the ability to degrade *p*-nitrophenol (PNP). Cellular fatty acid analysis revealed that the predominant components were anteiso-C_15:0_ (24.61%), C_11:0_ (21.06%), iso-C_16:0_ (11.89%), C_16:0_ (11.58%), and anteiso-C_17:0_ (11.24%). Notably, the presence of C_11:0_, C_10:0_ 2-OH as major fatty acids differentiate strain DNPG3^T^ from its closely related members of the genus *Brachybacterium*. The predominant respiratory quinone was identified as menaquinone-7 (MK-7). Analysis of 16S rRNA gene sequence indicated that *B. zhongshanense* strain JB^T^ was the closest relative of DNPG3^T^, sharing 97.08% sequence similarity. Genome-based ANI value calculated using the EzBioCloud server revealed that *B. zhongshanense* JCM 15471^T^ was the closest genomic relative (85.49%). These values were further substantiated by digital DNA–DNA hybridization (dDDH) estimates calculated using the GGDC server. Taxonomic assignment using the GTDB database further indicated that strain DNPG3^T^ constitutes a previously unrecognized species within the genus *Brachybacterium*. Genome analysis of strain DNPG3^T^ identified eleven genomic islands, along with a rich repertoire of 194 carbohydrate-active enzyme (CAZyme) families, comprising 95 glycoside hydrolases and 53 glycosyltransferases. In addition, five biosynthetic gene clusters were detected. Collectively, these genomic features indicate the involvement of horizontal gene transfer events and highlighted the pronounced metabolic versatility of the strain, underscoring its potential for industrial enzyme production and secondary metabolite biosynthesis. Pan-genome analysis further indicates that the *Brachybacterium* pan-genome is open, reflecting substantial genetic diversity and ongoing gene acquisition within the genus. Comprehensive biochemical, physiological, chemotaxonomic, and phylogenetic analyses supported the assignment of strain DNPG3^T^ to the genus *Brachybacterium* while clearly distinguishing it from all currently described species within the genus. Accordingly, strain DNPG3^T^ was proposed to represent a novel species, for which the name *Brachybacterium netajii* sp. nov. is suggested. The type strain was DNPG3^T^ (= MTCC13125^T^).

**Supplementary Information:**

The online version contains supplementary material available at https://doi.org/10.1038/s41598-026-56775-0.

## Introduction

The family Dermabacteraceae of phylum Actinobacteria encompasses several genera, notably *Brachybacterium*, *Dermabacter*, *Devriesea*, and *Helcobacillus*. Within this family, the genus *Brachybacterium*, first established by Collins et al*.*^1^*,* is currently represented by 27 species with validly published names, along with eight additional species not yet validly described (https://lpsn.dsmz.de/genus/brachybacterium on 12th January, 2026)^2^. Members of the genus *Brachybacterium* are Gram-positive, non–acid-fast, non-motile, and non-endospore-forming bacteria. They have been isolated from a broad spectrum of ecological niches, reflecting their notable environmental adaptability. Reported habitats include river sediments^3^, salt-fermented seafood^4^, the liver of laboratory mice^5^, oil-contaminated coastal sands^6^, Atlantic ocean^7^, human blood culture^8^ and garden soil^9^.

To date, considerable functional diversity has been reported within the genus *Brachybacterium*. Among the described species, *B. phenoliresistens* is the sole member reported to exhibit resistance to xenobiotic compounds^6^, while the capacity for heavy-metal bioremediation, particularly manganese, has been demonstrated in *Brachybacterium* sp. strain Mn32^10^. Biodegradation of PNP has not been documented for any member of the genus, with the single exception of *Brachybacterium* sp. strain DNPG3^11^. *Brachybacterium* sp. UFH, isolated from the freshwater environment of South Africa, has been reported to possess significant bioflocculant-producing potential^12^. Thus, the metabolic versatility exhibited by *Brachybacterium* strains underscores the significance of contaminated river water as a valuable reservoir of bacterial metabolites with considerable biotechnological potential. Consequently, the isolation and characterization of novel strains from such environments are of paramount importance.

In this study, we present the taxonomic characterization of a novel PNP–degrading strain, DNPG3^T^, employing a comprehensive polyphasic approach. Integrated analyses encompassing phenotypic and chemotaxonomic characteristics, 16S rRNA gene–based phylogeny, FAME profiling, major menaquinone composition, and whole-genome sequencing collectively demonstrate that strain DNPG3^T^ represents a previously unrecognized species within the genus *Brachybacterium*. Accordingly, the name *Brachybacterium netajii* sp. nov. is proposed for this strain.

## Materials and methods

### Bacterial isolation, characterization, and heavy metal tolerance

Water samples were aseptically collected from the river Ganges in W.B, India (23°52′59″ N, 88°24′05″ E). Isolation of bacterial strain DNPG3^T^ was performed using an enrichment culture technique in minimal medium (MM) supplemented with 0.25 mM 2,4-dinitrophenol (DNP), followed by PNP as the sole carbon source, following established protocols^11^. When required, the basal minimal medium was solidified with 1.5% (w/v) agar. Growth of the bacterial strains on MM supplemented with PNP and/or decolorization of the characteristic yellow medium were taken as indicative of a positive PNP biodegradation phenotype. Phenotypic characterization of the strain was performed in accordance with the methods described by Smibert and Krieg^13^.

Strain DNPG3^T^ was cultured in tryptone soy broth, harvested during the exponential growth phase, and thoroughly washed with basal minimal medium (MM). The cell density was adjusted to approximately 10⁷ cells mL⁻^1^ to obtain a standardized inoculum for subsequent growth assays. Heavy metal tolerance was assessed by supplementing the growth medium with selected water-soluble metal salts. Sodium arsenate (NaHAsO₄), cadmium chloride hexahydrate (CdCl₂·6H₂O), lead nitrate [Pb(NO₃)₂], and mercuric chloride (HgCl₂) were evaluated at concentrations ranging from 200 to 2000 ppm. Sucrose low-phosphate (SLP) broth was used as the basal medium and comprised 1% sucrose, 0.05% MgSO₄·7H₂O, 0.01% NaCl, 0.1% (NH₄)₂SO₄, and 0.05% yeast extract, with the pH adjusted to 7.0. Aliquots of the standardized inoculum (≈10⁷ cells mL⁻^1^) were inoculated into SLP broth containing varying metal concentrations and incubated at 28 °C for 72 h. The MIC was defined as the lowest metal concentration resulting in minimal viable cell counts, following the method described by Alam and Saha^14^.

Menaquinones were isolated from 200 mg of freeze-dried cell biomass using a solvent system comprising methanol containing 10% aqueous 0.3% (w/v) NaCl and petroleum ether mixed in a 1:1 (v/v) ratio. Following phase separation, the upper layer was collected and evaporated to dryness using a TurboVap LV evaporator. The resulting residue was re-dissolved in 0.1 ml of acetone. The extract was subsequently subjected to TLC on silica gel 60 F₂₅₄ plates, employing petroleum ether:diethyl ether (85:15, v/v) as the mobile phase^15^. A *Bacillus subtilis* strain, known to contain menaquinone-7 as the predominant quinone, was included as a positive control for comparative analysis.

For whole-cell fatty acid profiling, strain DNPG3^T^ was obtained from a 24-h culture grown on TSA at 37 °C. Cellular fatty acids were released by saponification, converted to methyl esters, and subsequently extracted following the standardized protocol of the MIDI Sherlock Microbial Identification System (MIDI ID No. 1408). The analysis was performed by Royal Life Sciences Pvt. Ltd., an ISO 9001:2015–certified laboratory affiliated with MIDI Sherlock, USA. FAMEs were separated and quantified using gas chromatography (Agilent GC-6850, USA), and individual fatty acids were identified by comparison with the ACTIN6 database of the Microbial Identification System.

### Phylogenetic analysis based on the 16S rRNA gene sequence

For bacterial identification, a molecular phylogenetic approach based on the 16S rRNA gene was employed. Genomic DNA was extracted following the protocol described by Pitcher *et al*^16^. The nearly complete 16S rRNA gene was amplified from the genomic DNA of DNPG3^T^ using the universal bacterial primers 27 F and 1492R. PCR amplification, thermal cycling conditions, and sequencing procedures were performed as described by Saha and Chakrabarti^17–19^.

Phylogenetic affiliation, including identification of closely related taxa and determination of pairwise sequence similarities, was assessed using BLAST and the EzTaxon server. Phylogenetic trees were prepared using the maximum-likelihood and neighbor-joining methods implemented in MEGA version 11, with bootstrap analysis based on 1,000 replicates to evaluate branch support. The obtained 16S rRNA gene sequence was deposited in the GenBank database under the accession number MW064125.

### Genome sequencing, annotation, and genome-based phylogenetic analysis

Genomic DNA of strain DNPG3^T^ was quantified using the Qubit 3 Fluorometer with the dsDNA HS assay kit. The library was purified using AMPure beads and enriched by PCR amplification (6 cycles) using the NEBNext Ultra II Q5 Master Mix, Illumina universal primers, and sample-specific index primers. The amplified library was further purified using AMPure beads and eluted in 15 μL of 0.1 × TE buffer. Library concentration was determined using the Qubit 3 Fluorometer, and fragment size distribution was assessed using the Agilent 2100 Bioanalyzer with a DNA 7500 chip.

The genomic DNA was sequenced using the Illumina NovaSeq 6000 platform, generating paired-end reads (2 × 150 bp) using Illumina sequencing-by-synthesis chemistry. De novo genome assembly was performed using the SPAdes assembler implemented in Unicycler version 0.5.1.1^20^. Genome completeness was assessed using BUSCO v6.0.0 with the *micrococcales_odb12* lineage dataset (504 single-copy orthologs), under the prokaryotic genome mode. In addition, the CheckM2 server was employed to assess genome completeness and detect potential contamination. Structural and functional annotation of the draft genome was conducted using multiple prokaryotic annotation pipelines, including the NCBI’s PGAP^21^, BAKTA^22^, RAST^23^ and PROKKA^24^. The CGView server was employed to generate a comprehensive circular graphical representation of the complete genome of strain DNPG3^T^^25^.

The ANI (Average nucleotide identity) analysis was performed using genome assemblies, with pairwise ANI values computed through the online IPGA platform (v1.09)^26^, ANI calculator (https://www.ezbiocloud.net/)^27^. For ANI analysis, genome of type strains: *Brachybacterium alimentarium* strain CCUG50349^T^ (JBHSLP010000000), *B. aquaticum* strain DSM28796^T^ (JACHLZ010000000), *B. atlanticum* strain PhyBa_CO2_2^T^ (JAKJJW000000000), *B. avium* strain VR2415^T^ (CP022316), *B. conglomeratum* strain_CCM2589^T^ (JBHLYZ010000000), *B. endophyticum* strain M1HQ-2^ T^ (QFKX01000001.1), *B. epidermidis* strain Marseille-Q2903^T^ (JADEYR000000000), *B. equifaecis* strain JHP9^T^ (JAKNCJ010000000), *B. faecium* DSM 4810^ T^ (CP001643.1), *B. fresconis* strain CCUG50350^T^ (JBHSLO010000000), *B. ginsengisoli* strain DCY80^T^ (CP023564.1), *B. hainanense* strain CICC_10874 (JBHLSV010000000), *B. halotolerans* strain MASK1Z-5^ T^ (JAEDAJ010000000), *B. huguangmaarense* strain BRM-3 (CP107020.1), *B. kimchii* strain CBA3104^T^ (CP097218.1), *B. massiliense* strain MT5^T^ (FXXB00000000), *B. muris* strain CCM_7047^T^ (JBHLVL010000000), *B. nesterenkovii* strain CIP104813^T^ (FWFG00000000), *B. paraconglomeratum* strain KCTC 9916 (ML133851), *B. phenoliresistens* JCM15157^T^ (BAAAOW010000000), *B. rhamnosum* JCM11650^T^ (BAAAIS010000000), *B. sacelli* strain CCUG50347^T^ (JBHSLQ010000000), *B. saurashtrense* strain DSM 23186^ T^ (CP031356), *B. sillae* strain EF45031^T^ (JAFEUW010000000), *B. squillarum* strain M-6-3^ T^ (AGBX01000000), *B. subflavum* strain CFH10395^T^ (VIGP01000000), *B. timonense* strain Marseille P4339^T^ (LT984895), *B. tyrofermentans* strain CCUG50353^T^ (JBHSLN010000000), *B. vulturis* strain VM2412^T^ (CP023563), *B. zhongshanense* strain JCM15471^T^ (BBHA01000000) were retrieved from the NCBI Genome database.

Species-level relatedness was assessed by applying the widely accepted ANI cutoff of approximately 95%. In parallel, dDDH values were estimated using the GGDC 3.0 server (accessed on 30 November 2025)^28^. In accordance with established bacterial taxonomic standards, a dDDH value of ~ 70% was used as the benchmark for species delineation. Species-level identification was further performed using the Type (Strain) Genome Server (TYGS;https://tygs.dsmz.de)^29^. Whole-genome assemblies were uploaded to the TYGS platform, where phylogenomic relationships were inferred using the Genome BLAST Distance Phylogeny (GBDP) methodology. Pairwise digital dDDH estimates were generated by comparison with reference type strain genomes, and taxonomic assignments were determined based on the established species boundaries. Genus identification of DNPG3^T^ was carried out in the KBase platform using the GTDB-Tk workflow, wherein whole-genome phylogenetic placement analyses was employed to assign the strain to its appropriate genus according to the Genome Taxonomy Database (GTDB)^30^.

### Genomic island and CAZymes prediction

Genomic islands (GIs) are large, discrete DNA regions within bacterial genomes that are primarily acquired through horizontal gene transfer rather than through vertical inheritance. These elements are key drivers of bacterial evolution and ecological diversification, as they facilitate the acquisition of adaptive traits that enhance survival and competitiveness within specific niches. In pathogenic bacteria, GIs are often enriched in genes encoding virulence determinants and antimicrobial resistance, thereby contributing disproportionately to pathogenicity and fitness^31^. In addition, GIs frequently harbor novel genes associated with niche-specific functions, including heavy metal resistance and complete metabolic pathways that expand the organism’s functional repertoire. In the present study, the genomic islands present in the genome of strain DNPG3^T^ and a few close relatives of the strain were characterized using the IslandViewer4 platform^32^.

To identify and characterize the repertoire of carbohydrate-active enzymes (CAZymes), the predicted protein sequences of strain DNPG3^T^ were functionally annotated using the dbCAN2 meta-server^33^.

### Pan genome

The pan-genome and core genome of *Brachybacterium* were analyzed using IPGA v1.09 server with default parameters, following the methodology described by Liu et al*.*^26^.

### Secondary metabolite biosynthetic gene cluster analysis

Putative secondary metabolite biosynthetic gene clusters were identified using the antiSMASH platform (version 7.0; accessed on 22 December 2025). The analysis was conducted with default parameters and a relaxed detection strictness to enable comprehensive cluster prediction. antiSMASH facilitates reliable identification of known and putative secondary metabolite gene clusters through the application of curated profile hidden Markov models specifically designed for biosynthetic pathway recognition^34^.

## Results and discussion

### Isolation, characterization, and heavy metal tolerance

The bacterial strain DNPG3^T^ was isolated from water samples collected from the River Ganges using an enrichment culture approach, with PNP or DNP provided as the sole carbon sources. Strain DNPG3^T^ was initially enriched and selectively isolated using DNP as the exclusive carbon source, indicating its metabolic capability to utilize xenobiotic compounds under selective environmental pressure. Preliminary growth-based assays demonstrated that strain DNPG3^T^ effectively decolorized the yellow coloration of 0.3 mM PNP within 48 h of incubation at 30°C^1^ (Supplementary Fig. 1). The MIC of the strain for PNP was up to 3 mM, while maximal growth was observed at 0.25 mM PNP^1^. The strain exhibited a high level of heavy metal tolerance, withstanding concentrations of up to 1000 ppm sodium arsenate (NaHAsO₄), 1200 ppm cadmium chloride (CdCl₂·6H₂O), 600 ppm lead nitrate [Pb(NO₃)₂], and 1400 ppm mercuric chloride (HgCl₂). TLC analysis revealed that strain DNPG3^T^ possesses menaquinone-7 (MK-7) as its predominant respiratory quinone. The TLC profile was comparable to that of the reference strain *Bacillus subtilis*, confirming the dominance of MK-7 in DNPG3^T^. The presence of MK-7 as the major menaquinone is consistent with its taxonomic affiliation and supports its placement within related Gram-positive bacterial lineages (Supplementary Fig. 2). Cellular fatty acid profiling of strain DNPG3^T^ revealed anteiso-C_15:0_ (24.61%) as the dominant component, followed by C_11:0_ (21.06%), iso-C_16:0_ (11.89%), C_16:0_ (11.58%), and anteiso-C_17:0_ (11.24%). Notably, the occurrence of C_11:0_, C_10:0_ 2-OH, and C_9:0_ fatty acids distinguish strain DNPG3^T^ from its closest phylogenetic relatives within the genus *Brachybacterium*. A comparative analysis of the cellular fatty acid compositions of strain DNPG3^T^ and related taxa is presented in Table 1.

**Table 1:** Cellular fatty acid composition of strain DNPG3^T^ and its phylogenetically related species of the genus *Brachybacterium.*

	**Percentage of total fatty acid (%)**				
**Type of fatty acids**	***Brachybacterium netajii* strain DNPG3**^**T**^	***B. zhongshanense*** **strain JB**^**T**^	***B. muris*** **strain C3H-21**^** T**^	***B. squillarum*** **strain M-6-3**^** T**^	***B. horti*** **strain THG S15-4**^** T**^
Straight-chain fatty acids					
C_9:0_	2.44	-	-	-	-
C_10:0_	1.36	-	-	-	-
C_10:0_ 2OH	4.79	-	-	-	-
C_11:0_	21.06	-	-	-	-
C_14:0_	0.80	1.42	-	-	1.1
C_16:0_	11.58	2.23	1.0	-	6.6
C_18:0_	0.57	-	-	-	2.2
C_19:0_	-	-	5.3	-	-
Branched-chain fatty acids					
C_11:0_ iso	1.07	-	-	-	-
C_14:0_ iso	1.09	1.45	5.7	7.0	5.0
C_15:0_ iso	5.66	9.22	4.5	5.4	2.0
C_15:0_ anteiso	24.61	37.38	60.30	62.1	51.5
C_16:0_ iso	11.89	13.13	7.2	13.2	16.3
C_17:0 i_so	0.84	-	-	-	-
C_17:0_ anteiso	11.24	28.17	5.3	12.3	6.6
Unsaturated fatty acids					
C_18:1_ ω7c	-		4.0	-	-
C_20:1_ ω7c	-	2.20	-	-	1.6

The strain was determined to be Gram-positive, exhibited tolerance to NaCl concentrations of up to 9% (w/v), and showed positive starch hydrolysis activity. The comparative phenotypic characteristics of strain DNPG3^T^ in relation to its closest phylogenetic relatives are summarized in Table 2. The strain exhibited positive reactions for β-glucosidase activity and demonstrated the ability to utilize a range of carbohydrates, including sucrose, D-glucose, D-maltose, D-mannose, D-trehalose, D-cellobiose, D-sorbitol, and D-mannitol (enzyme-based). In addition, DNPG3^T^ showed positive responses for adenosine assimilation, L-lactate (kinetic) utilization, and citrulline metabolism. The strain also displayed γ-glutamyl transferase and cysteine arylamidase activities, along with a positive oxidative/fermentative reaction, indicating metabolic versatility and active participation in carbohydrate and amino-acid turnover.

**Table 2:** Comparative analysis of the phenotypic characteristics of strain DNPG3^T^ and its closely related species within the genus *Brachybacterium.*

	***Brachybacterium netajii* strain DNPG3**^**T**^	***B. Zhongshanense*** **strain JB**^**T**^	***B. muris* strain C3H-21**^** T**^	***Brachybacterium squillarum* strain** **M-6-3**^** T**^	***Brachybacterium horti* strain** **THG S15-4**^** T**^
pH Tolerance	5.8–8	5–10	6–10	6.0–9.0	6.0–7.5
Habitat	River water, W.B, India	Sediment, China	liver of mouse	seafood in Korea	garden soil, Korea
NaCl tolerance	Upto 9%	Upto 10%	Upto 10%	Upto 10%	Upto 2%
Optimum Temperature	30 °C	30 °C	25 °C-37°C	28 °C-30°C	28 °C
p-nitrophenol degrading ability	P, Upto 3 mM	ND	ND	ND	ND
Gram’s Reaction	Gram Variable	Gram Positive	Gram Positive	Gram Positive	Gram Positive
Catalase Test	P	P	P	N	P
Citrate Test	N	ND	ND	N	N
MR Test	P	ND	ND	ND	ND
Indole Test	N	N	N	N	N
VP Test	N	N	N	N	ND
Oxidase Test	N	P	N	N	P
Phosphate Solubilization	N	ND	ND	ND	ND
Starch hydrolysis	P	N	P	ND	N
H_2_S production	N	N	N	N	ND
Casein hydrolysis	N	ND	N	ND	N
Pectin Hydrolysis	N	ND	ND	ND	ND
Chitin Hydrolysis	N	ND	ND	ND	N
Cellulose Hydrolysis	N	P	ND	ND	N
Tween 20	N	N	P	N	P
MacConkey agar growth	N	ND	ND	ND	N
GC content (%)	72.44	71.2	ND	71.5	69.5
D-xylose	P	N	N	N	ND
D-glucose	P	P	P	N	P
L-arabinose	P	P	P	N	ND
D-mannose	P	P	P	P	P
D-melibiose	P	P	N	N	ND

(P = positive; N = negative).

In contrast, strain DNPG3^T^ was negative for a wide array of enzymatic activities and carbon source utilization, including Ala-Phe-Pro arylamidase, pyrrolidonyl arylamidase, glycine arylamidase, proline arylamidase, β-alanine arylamidase, and gly-gly arylamidase, indicating limited peptide hydrolysis for these substrates. The strain did not produce hydrogen sulfide and lacked β-N-acetylglucosaminidase, lipase, phosphatase, *β*-glucuronidase, *α*-galactosidase, *α*-glucosidase, *β*-galactosidase, and* β*-xylosidase activities, reflecting restricted polysaccharide and glycoside degradation pathways. Additionally, DNPG3^T^ did not utilize D-tagatose, 5-keto-D-gluconate, succinate, palatinose (*α*-D-glucopyranosyl-1,6-fructose), N-acetyl-D-glucosamine, L-malate, L-arabinitol (non-enzyme-based), and L-lactate (non-kinetic). The strain was also negative for *α*-glucoside transport, ornithine decarboxylase, urease, tyrosine arylamidase, L-histidine arylamidase, and the Ellman reaction for thiol detection.

Collectively, these biochemical characteristics define a highly distinctive phenotypic profile that differentiates strain DNPG3^T^ from the currently described taxa in commercial identification databases. The observed metabolic breadth, coupled with selective substrate utilization and enzymatic specialization, supports the hypothesis that DNPG3^T^ represents a taxonomically distinct lineage.

### 16S rRNA gene-based identification

Preliminary BLASTN analysis of the 16S rRNA gene sequence revealed that *Brachybacterium* sp. strain DNPBRP2 (GenBank accession OP459200), another isolate obtained from the same environmental sample by our research group, was the closest phylogenetic relative of strain DNPG3^T^, exhibiting 98.67% sequence similarity. This was followed by *Brachybacterium* sp. strain DNPG4 (MW073529), also isolated and deposited by our group, which shared 98.45% 16S rRNA gene sequence similarity. Among the validly published type strains, *Brachybacterium zhongshanense* strain JB^T^ was identified as the closest phylogenetic relative of strain DNPG3^T^, exhibiting 97.08% 16S rRNA gene sequence identity, followed by *B. atlanticum* strain PhyBa_CO2_2^T^ with 96.70% sequence similarity (Supplementary Table 1). The threshold value for 16S rRNA gene sequence similarity used in bacterial species delineation has been progressively refined. While an earlier widely accepted cut-off value was 97%^35^, subsequent studies proposed a more stringent threshold of 98.65% for species-level identification^36^. In the present study, strain DNPG3^T^ exhibited a 16S rRNA gene sequence similarity of 97.08% with its closest type strain, *B. zhongshanense* JB^T^, which is significantly below the currently accepted threshold of 98.65%. Therefore, strain DNPG3^T^ may represent a potential novel species within the genus *Brachybacterium*.

**Fig. 1:**
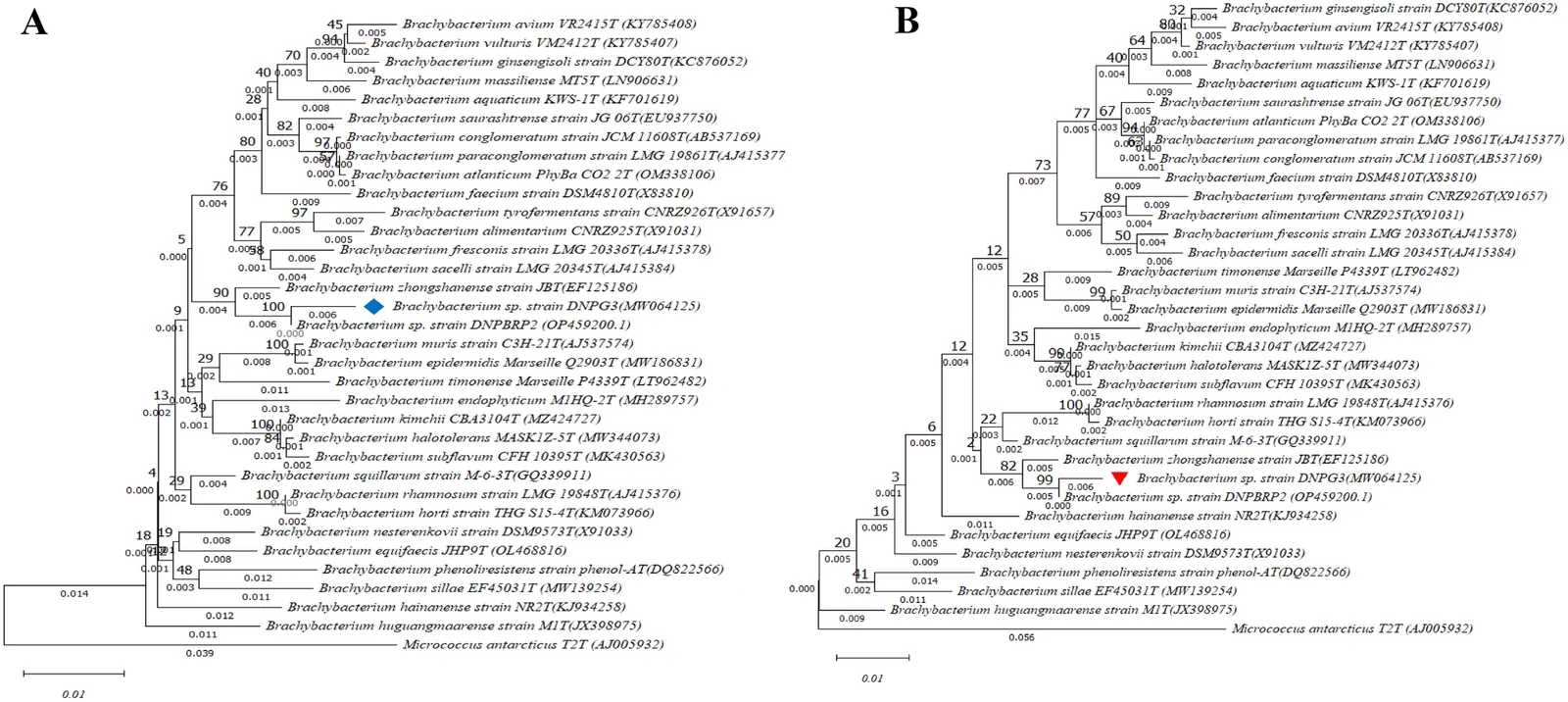
(**A**) Neighbor-joining and (**B**) ML phylogenetic trees based on 16S rRNA gene sequence analysis illustrated the phylogenetic placement of strain DNPG3 among type strains of the genus *Brachybacterium*. Bootstrap values, expressed as percentages from 1,000 replicates, are indicated at the branch nodes. *Micrococcus antarcticus* T2^T^ was employed as the outgroup.

The phylogenetic position of strain DNPG3^T^ was determined through comparative analysis of its 16S rRNA gene sequence using the neighbor-joining (NJ) method implemented in MEGA version 11. In the resulting phylogram, strain DNPG3^T^ clustered closely with *Brachybacterium* sp. strain DNPBRP2, which was previously recovered from the same ecological niche, suggesting a close evolutionary relationship between these isolates. This association was supported by a maximum bootstrap value of 100%, indicating strong statistical confidence in the inferred grouping. Among the validly described type strains, *B. zhongshanense* JB^T^ was identified as the closest phylogenetic relative of DNPG3^T^ (Fig. 1A). Nevertheless, the observable branch length separating DNPG3^T^ from its nearest type strain neighbors reflects a discernible level of evolutionary divergence. Collectively, the 16S rRNA gene-based neighbor-joining phylogeny substantiates the placement of strain DNPG3^T^ within the genus *Brachybacterium*, while also emphasizing its genetic distinctiveness from currently recognized species.

In agreement with the NJ analysis, the maximum-likelihood (ML) phylogeny placed strain DNPG3^T^ unambiguously within the *Brachybacterium* clade, confirming its generic affiliation. Strain DNPG3^T^ formed a distinct, well-supported lineage in close association with *Brachybacterium* sp. strain DNPBRP2. Among the validly published type strains, *B. zhongshanense* JB^T^ emerged as the nearest phylogenetic relative (Fig. 1B). Overall, the ML reconstruction corroborated the NJ topology and further supported the conclusion that strain DNPG3^T^ represents a genetically distinct lineage within the genus *Brachybacterium*.

### Genome features and genome‑based phylogeny

BUSCO v6.0.0 analysis indicated that the genome assembly exhibited 96.6% completeness, including 96.4% complete single-copy BUSCOs and 0.2% duplicated BUSCOs, with 0.6% fragmented BUSCOs and 2.8% missing BUSCOs. The very low proportion of duplicated BUSCOs suggests minimal potential contamination in the genome assembly. Whereas the CheckM2 analysis indicated a genome completeness of 99.99%, with an extremely low level of contamination (0.05%), reflecting high-quality assembly and reliability of the dataset.

To precisely resolve the phylogenetic position of the proposed novel species and assess its genomic distinctiveness, dDDH, ANI, and genome-to-genome distance analyses were performed. The genome of strain DNPG3^T^ comprised a total of 3,217 genes, including 3,157 protein-coding sequences (CDSs) and 25 pseudogenes. Additionally, the genome encodes 52 tRNA genes, 5 rRNA genes, and 3 non-coding RNA (ncRNA) genes. The assembled genome comprised contigs ranging in length from 374 bp to 876,926 bp, with an overall average G + C content of 72.5%. RAST-based annotation revealed that 22% (717) of the predicted protein-coding genes were assigned to 240 functional subsystems (Supplementary Fig. 3). Among these, genes involved in the metabolism of amino acids and their derivatives were the most abundant (207), followed by those associated with carbohydrate metabolism and protein metabolism (161 each). Additionally, a notable subset of genes was related to stress response (13) and the metabolism of aromatic compounds (2).

**Fig. 2:**
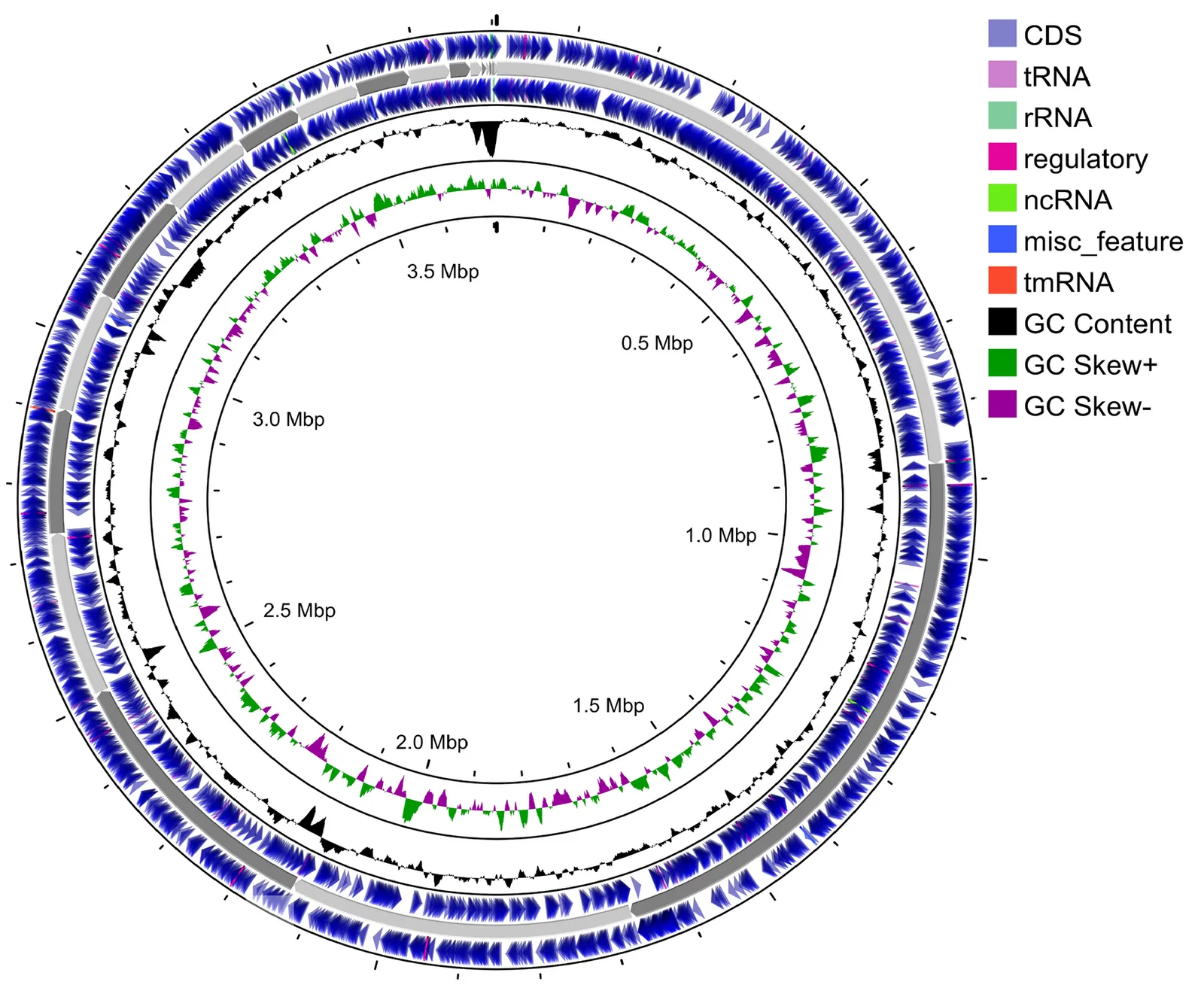
A circular genome map illustrating the genomic features of strain DNPG3 was generated using the CGView server.

The circular genome map of strain DNPG3^T^, generated using the CGView platform (Fig. 2), provided an integrated and holistic visualization of its genomic structure, functional organization, and compositional characteristics. The outermost concentric rings depicted the distribution of predicted CDSs on the forward and reverse strands, revealing a dense and largely uniform gene arrangement throughout the genome. Such even gene distribution indicates a compact and efficiently organized bacterial genome. Inner rings representing tRNA and rRNA genes illustrated the genomic positioning of essential components of the translational machinery. The widespread occurrence of multiple tRNA genes across the genome supported robust and efficient protein synthesis, whereas the comparatively limited and conserved rRNA operons reflect the stability of core housekeeping functions. In addition, the visualization of ncRNAs underscored the regulatory complexity of strain DNPG3^T^, facilitating precise control of gene expression in response to environmental and physiological conditions. The GC content ring revealed notable fluctuations across the genome, consistent with the GC-rich nature of the DNPG3^T^ genome. Localized deviations in GC content may signify regions acquired via horizontal gene transfer, such as genomic islands. These regions frequently harbor genes associated with adaptive functions, including stress resistance, specialized metabolic capabilities, and defense mechanisms.

Furthermore, the GC skew plots (positive and negative) provided insights into replication dynamics and chromosomal polarity. Collectively, the circular genome map of strain DNPG3^T^ highlighted the evolutionary stability of the core genome while maintaining flexibility through accessory regions that enhance ecological fitness and niche adaptation.

Functional annotation performed using the RAST and PGAP pipelines revealed a diverse repertoire of membrane transport systems in strain DNPG3^T^, reflecting its capacity for ion homeostasis and environmental adaptation. These included an extensive copper transport system comprising 11 genes, magnesium transporters encoded by two genes, and a multi-subunit cation/proton antiporter system represented by seven genes. The ability of strain DNPG3^T^ to tolerate high salinity levels, up to 9% NaCl, is well supported by the genomic presence of multiple osmotic stress–associated genes (six genes). In addition, genes involved in choline and betaine uptake, as well as betaine biosynthesis (four genes), were identified, highlighting the role of compatible solutes in maintaining cellular osmotic balance under saline conditions.

Furthermore, the genome of strain DNPG3^T^ encoded a broad array of proteolytic enzymes, including metalloproteases, serine proteases and cysteine proteases. The presence of these diverse protease classes suggests metabolic versatility and an enhanced capacity for protein turnover, nutrient acquisition, and environmental adaptability. Bacterial proteases constitute one of the most significant classes of industrial enzymes, with extensive applications across the leather, detergent, food, textile, and pharmaceutical sectors. In addition to their industrial utility, these enzymes play an important role in the bioremediation of industrial, domestic, and medical wastes by facilitating the degradation of complex proteinaceous materials^37^.

**Fig. 3:**
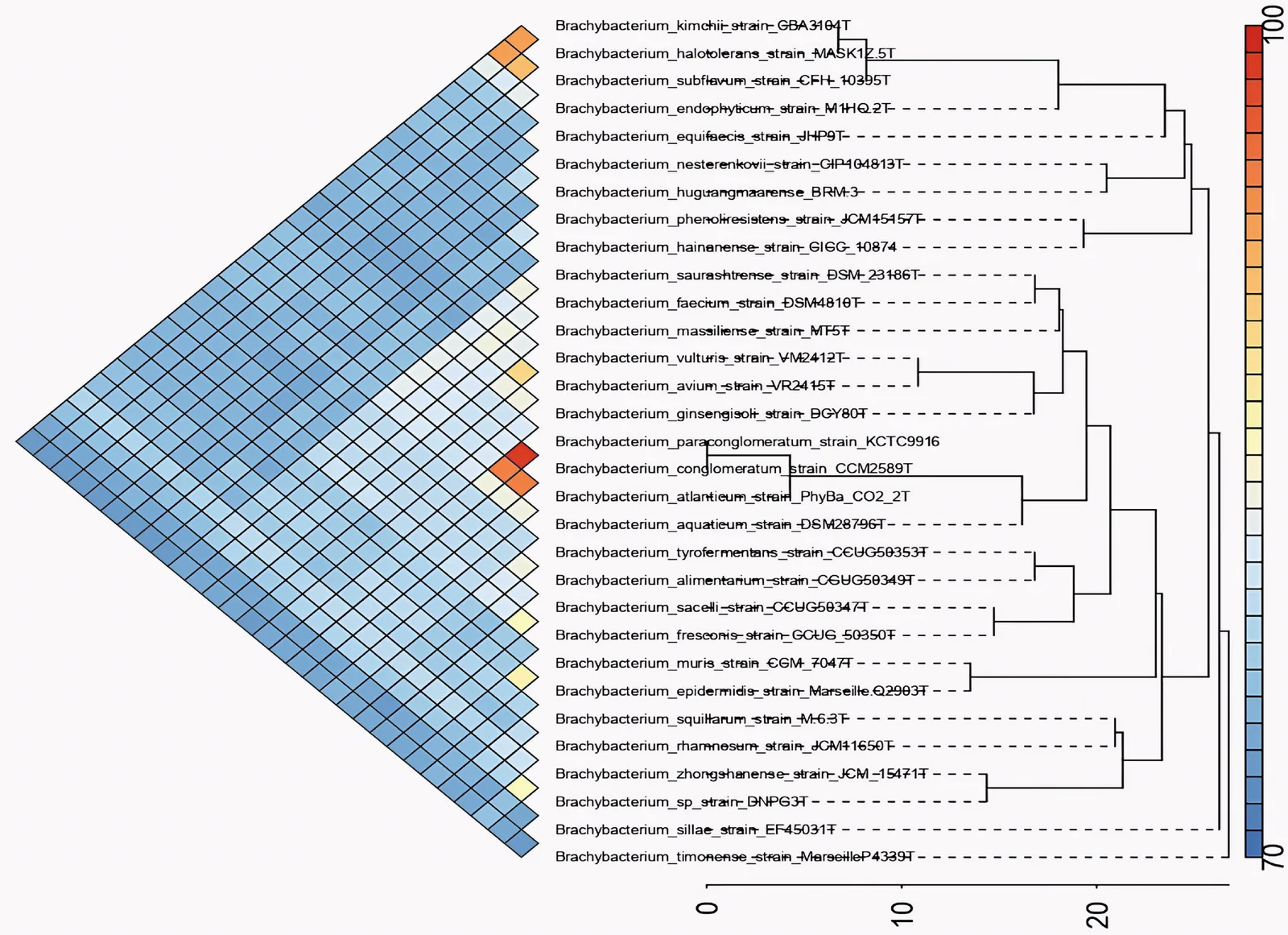
ANI analysis performed using IPGA v1.09 demonstrated that strain DNPG3 forms a clearly distinct genomic lineage, remaining well separated from all validly described type strains of the genus *Brachybacterium*.

In the IPGA v1.09 based heatmap, strain DNPG3^T^ formed a clearly distinct lineage, remaining well separated from all validly described *Brachybacterium* type strains (Fig. 3). The pairwise ANI values between DNPG3^T^ and its nearest phylogenetic neighbours were consistently below the widely accepted species demarcation threshold. Consequently, DNPG3^T^ did not cluster with any recognized species within the genus but instead branched independently, indicating substantial genome-level nucleotide divergence.

Pairwise ANI values between DNPG3^T^ and all examined reference genomes, conducted using the EZBioCloud ANI calculator, ranged from approximately 75.2% to 85.5%, which were well below the widely accepted species delineation threshold (Table 3). The highest ANI value was recorded with *Brachybacterium zhongshanense* JCM 15471^T^ (85.49%), followed by *B. squillarum* M6-3^T^ (78.84%), *B. conglomeratum* CCM 2589^T^ (78.83%), and *B. aquaticum* DSM 28796^T^ (78.46%). The uniformly low ANI scores obtained across both type and non-type reference strains demonstrated that DNPG3^T^ does not reside within the genomic boundaries of any previously described *Brachybacterium* species.

**Table 3:** Comparative analysis of ANI between strain DNPG3^T^ and its phylogenetically closely related species within the genus *Brachybacterium*.

	**Species of *Brachybacterium***	**ANI score**
DNPG3^T^	*Brachybacterium alimentarium* strain CCUG 50349^ T^	77.83
	*Brachybacterium aquaticum* strain DSM 28796^ T^	78.46
	*Brachybacterium atlanticum* strain PhyBa_CO2_2^T^	78.30
	*Brachybacterium avium* strain VR2415^T^	77.18
	*Brachybacterium conglomeratum* strain CCM 2589^ T^	78.82
	*Brachybacterium endophyticum* strain M1HQ-2^ T^	76.54
	*Brachybacterium faecium* strain DSM 4810^ T^	78.00
	*Brachybacterium fresconis* strain CCUG 50350^ T^	77.59
	*Brachybacterium hainanense* strain CICC 10874^ T^	76.73
	*Brachybacterium halotolerans* strain MASK1Z-5^ T^	76.96
	*Brachybacterium huguangmaarense* BRM-3 (not type strain)	76.39
	*Brachybacterium kimchii* strain CBA3104^T^	76.82
	*Brachybacterium muris* strain CCM 7047^ T^	77.14
	*Brachybacterium nesterenkovii* strain CIP104813 (not type strain)	76.41
	*Brachybacterium paraconglomeratum* strain KCTC 9916 (not type strain)	78.45
	*Brachybacterium phenoliresistens* strain JCM 15157^ T^	76.86
	*Brachybacterium rhamnosum* strain JCM 11650^ T^	78.73
	*Brachybacterium sacelli* strain CCUG 50347^ T^	77.65
	*Brachybacterium sillae* strain EF45031^T^	75.22
	*Brachybacterium saurashtrense* strain DSM 23186^ T^	77.91
	*Brachybacterium squillarum* strain M-6-3^ T^	78.84
	*Brachybacterium subflavum* strain CFH 10395^ T^	76.92
	*Brachybacterium tyrofermentans* strain CCUG 50353^ T^	77.99
	*Brachybacterium vulturis* strain VM2412^T^	77.60
	*Brachybacterium zhongshanense* strain JCM 15471^ T^	85.49
	*Brachybacterium timonense* strain Marseille-P4339^T^	75.42
	*Brachybacterium ginsengisoli* strain DCY80^T^	78.33
	*Brachybacterium epidermidis* strain Marseille-Q2903^T^	76.97
	*Brachybacterium equifaecis* strain JHP9^T^	76.37
	*Brachybacterium massiliense* strain MT5^T^	78.03

The dDDH analysis was carried out using the GGDC version 3.0 to quantitatively assess genome-wide relatedness between strain DNPG3^T^ and closely related members of the genus *Brachybacterium*. The dDDH is widely regarded as a benchmark approach for bacterial species circumscription, with a threshold value of 70% commonly applied for species-level assignment. In the GGDC output, higher dDDH values (typically depicted by green to yellow color gradients), are restricted to comparisons among strains of the same species, whereas lower values, predominantly shown in red, reflect substantial genomic divergence.

**Fig. 4:**
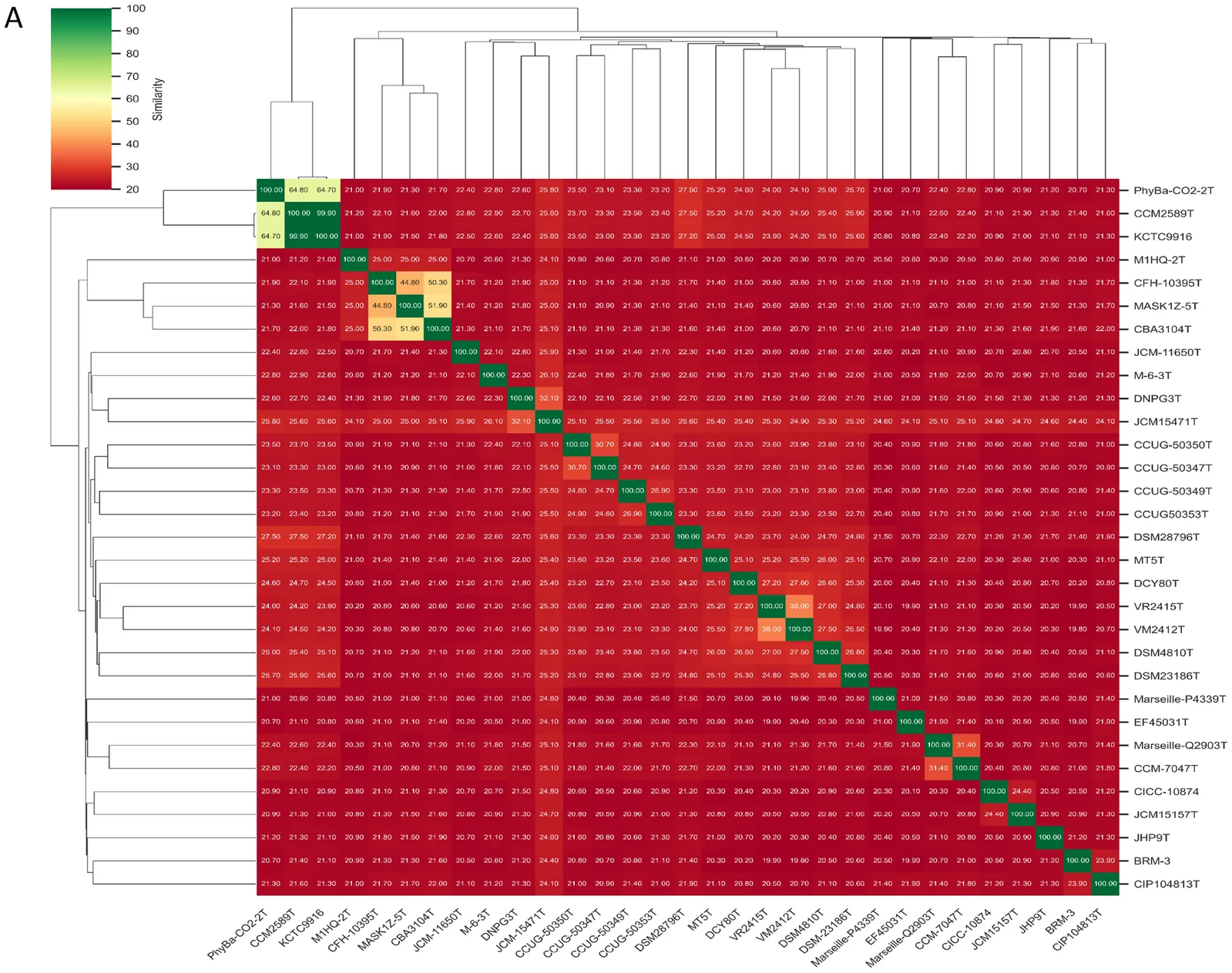

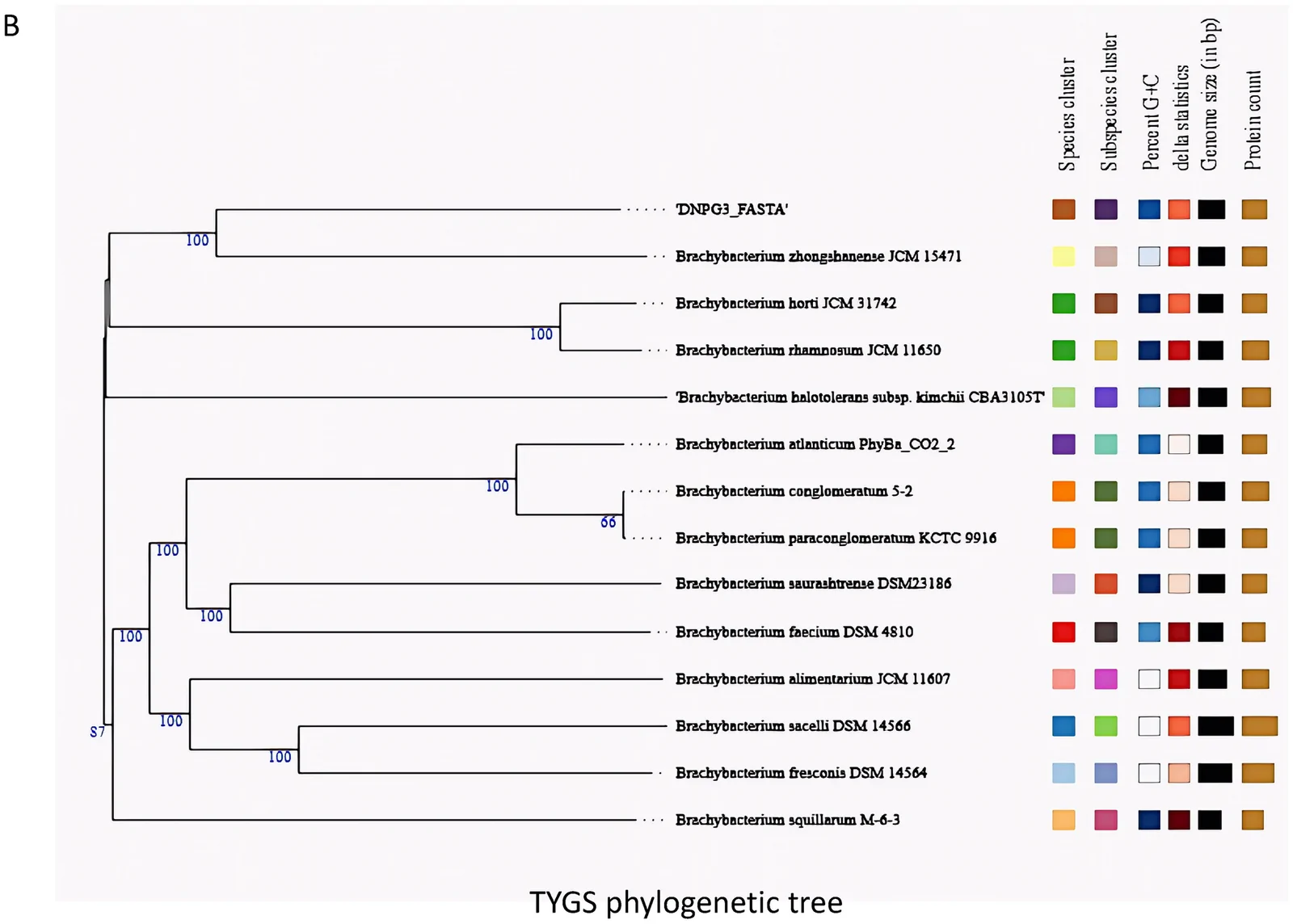

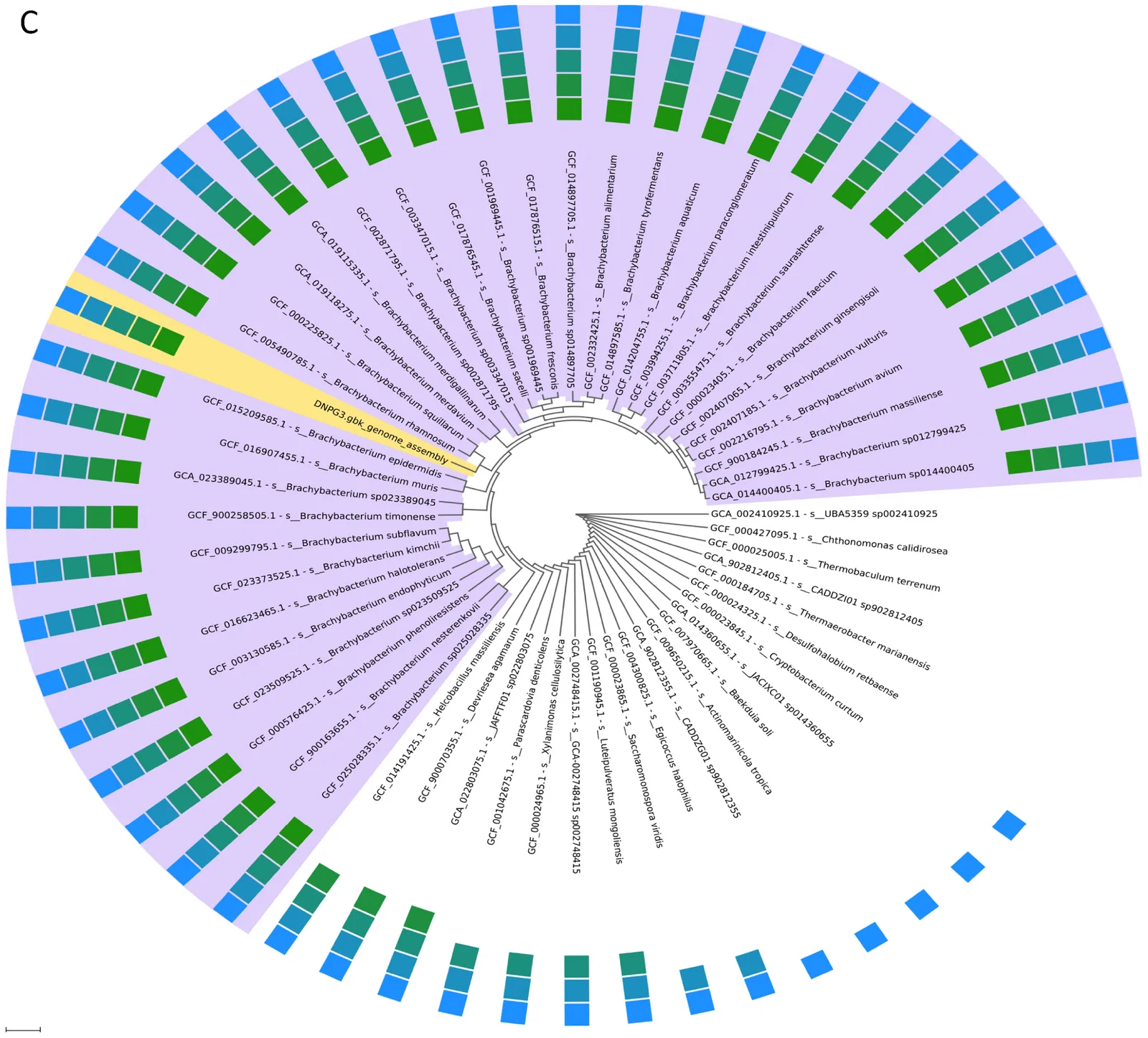
(**A**) dDDH analysis was performed using the GGDC version 3.0. (**B**) Phylogenetic tree construction was performed using TYGS based on whole-genome sequence data. (**C**) Phylogenetic tree based on GTDB indicated that strain DNPG3 represents a novel species within genus *Brachybacterium*.

Strain DNPG3^T^ consistently exhibited low dDDH values in comparison with all reference strains including *B. zhongshanense* JCM 15471^T^ showing the highest value (32.10%) which remaining well below the accepted 70% species boundary (Fig. 4A). Accordingly, DNPG3^T^ did not cluster with any recognized *Brachybacterium* species but instead formed an independent branch, indicative of pronounced genome-wide divergence from its closest phylogenetic relatives. This pattern was uniform across the dataset, suggesting that the divergence is not confined to discrete genomic segments but rather represents overall genomic dissimilarity. Collectively, ANI estimates derived from the EZBioCloud platform and IPGA, together with dDDH values obtained via GGDC, provide robust quantitative evidence that strain DNPG3^T^ represents a genomically distinct species within the genus *Brachybacterium*.

Phylogenomic analysis conducted using the TYGS server demonstrated that strain DNPG3^T^ constituted a distinct and strongly supported lineage within the genus *Brachybacterium* (Fig. 4B). The strain clustered most closely with *B. zhongshanense* JCM 15471^T^, sharing a common node supported by a 100% bootstrap value, indicative of a close evolutionary relationship. In addition, dDDH value between *B. zhongshanense* JCM 15471^T^ and strain DNPG3^T^, as calculated by the TYGS server, was 46.6%, which is well below the species delineation threshold (Supplementary Table 2). This level of genomic relatedness is consistent with previously reported dDDH values for novel species within the genus *Brachybacterium*, further supporting the recognition of strain DNPG3^T^ as a distinct species. Nevertheless, DNPG3^T^ was resolved as an independent branch rather than being embedded within the *B. zhongshanense* lineage, highlighting its genomic divergence and taxonomic distinctiveness.

In contrast, other type strains—including *B. horti JCM31742*^T^*, B. rhamnosum* JCM11650^T^, formed discrete and well-supported subclades with their established taxonomic assignments. The clear delineation of these lineages underscores the robustness and reliability of the phylogenomic reconstruction. The discrepancy between the GGDC 3.0 value (32.10%) and the TYGS value (46.6%) can be attributed to the use of different distance calculation formulas implemented within these platforms. Although both approaches are based on the GGDC methodology, the application of distinct formulas may generate varying dDDH estimates for the same pair of genomes.

Taxonomic assignment using the Genome Taxonomy Database (GTDB) further indicated that strain DNPG3^T^ represents a novel species within the genus *Brachybacterium*, thereby reinforcing the conclusions drawn from previous phylogenetic and genome-based analyses (Fig. 4C).

Taken together, these findings place strain DNPG3^T^ within the genus *Brachybacterium*, identify *B. zhongshanense* as its closest phylogenetic relative, and demonstrate its clear genomic distinctiveness, supporting its recognition as a novel species.

### Genomic island and CAZymes

**Fig. 5:**
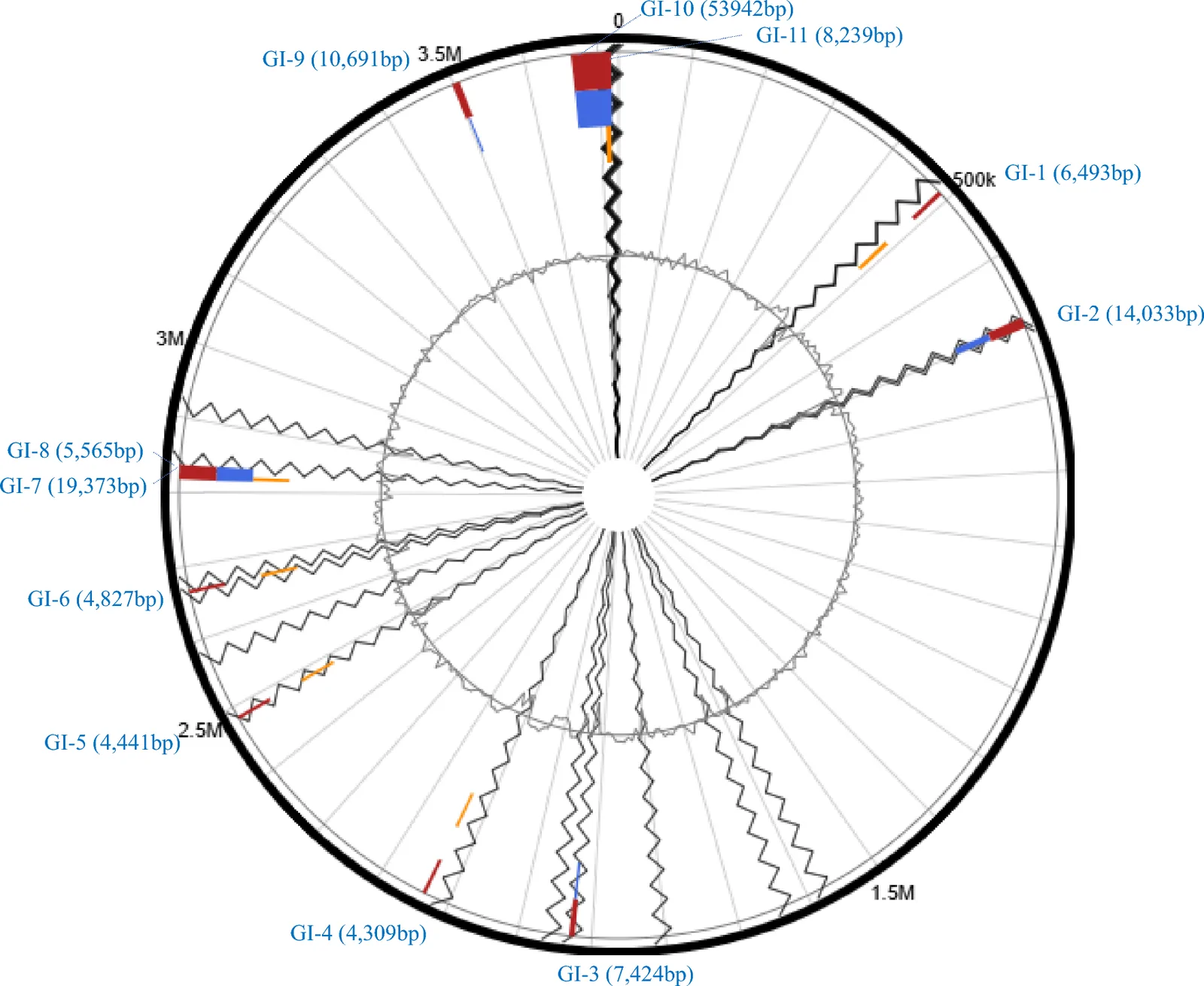
Genomic islands (GIs) within the DNPG3 genome were identified and visualized using the IslandViewer 4 platform.

A total of 11 genomic islands (GIs) were identified across the genome of strain DNPG3^T^, indicating the presence of multiple horizontally acquired genomic regions (Fig. 5). The majority of the predicted genomic islands in strain DNPG3^T^ encoded hypothetical proteins. Notably, GI-1 and GI-4 were enriched with genes encoding glycosyltransferases (GTs). Genomic islands GI-3 and GI-9 encoded transposases belonging to the IS3 family, whereas GI-10 and GI-11 harbored IS5 family transposases. Transposases are enzymes associated with mobile genetic elements that facilitate genome rearrangements and dissemination of genetic material through horizontal gene transfer (HGT). By enabling the mobilization and integration of functionally relevant genes, these elements contribute significantly to genomic plasticity and adaptive potential. Their frequent occurrence is therefore commonly associated with microorganisms thriving in stressful or extreme environments, where rapid genetic innovation confers a selective advantage^38^. Genomic island GI-10 of strain DNPG3^T^ encoded the Type I-E CRISPR-associated protein Cse1 (CasA), a key component of the Cascade surveillance complex that mediates sequence-specific immunity against invading phages and plasmids. The presence of this protein within GI-10 indicates the horizontal acquisition of adaptive immune functions, likely driven by sustained selective pressure imposed by foreign genetic elements in the environment^39^. Genomic island GI-6 encoded the arsenate reductase ArsC, an enzyme that catalyzes the reduction of arsenate [As(V)] to arsenite [As(III)]^40^. Overall, the genomic islands of strain DNPG3^T^ constitute prominent evolutionary hotspots that significantly contribute to metabolic flexibility, environmental stress resilience, and protective defense strategies, collectively enabling effective adaptation to fluctuating and challenging ecological conditions.

The in silico analysis carried out by dbCAN2 server predicted a rich repertoire of carbohydrate-active enzymes (CAZymes) encoded within the DNPG3^T^ genome. In total, 194 CAZyme families were identified, encompassing 95 glycoside hydrolases (GHs), 53 glycosyltransferases (GTs), 20 carbohydrate-binding modules with glycoside hydrolases (GHs + CBMs), 15 carbohydrate esterases (CEs), 5 carbohydrate-binding modules and 4 auxiliary activity (AA) enzymes (Supplementary Table 3). Collectively, this diverse CAZyme complement, predicted through in silico analysis highlights the potential of strain DNPG3^T^ as a promising source of enzymes for industrial applications, including biomass conversion, bioprocessing, and the production of value-added biochemicals.

### Pan genome analysis

**Fig. 6:**
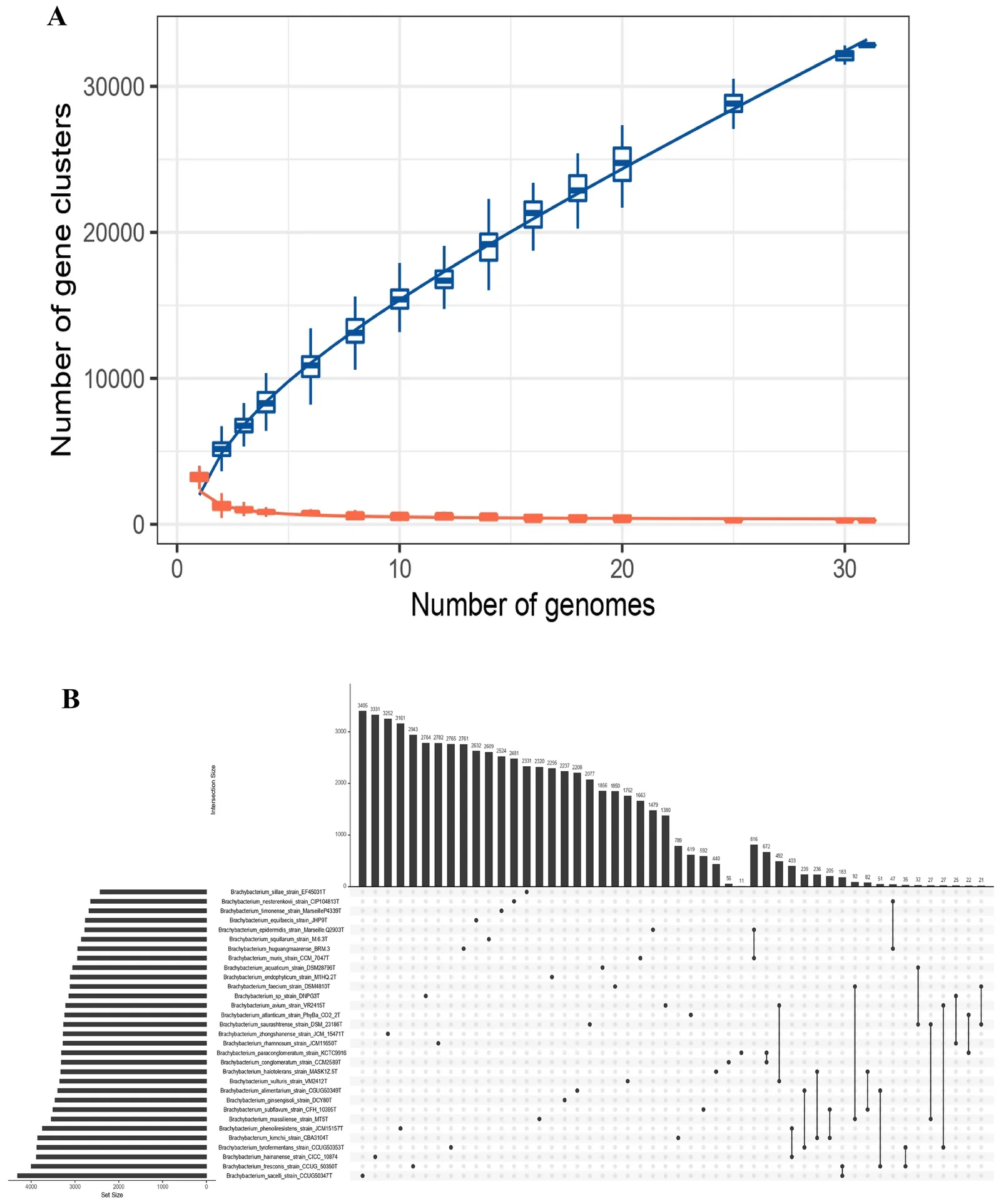
Pan genome analysis. (A) Distribution of pan-genome (blue) and core-genome (orange) clusters across various type strains of the genus *Brachybacterium*. (B) Upset plot illustrating the distribution and intersections of unique genes among type strains of the genus *Brachybacterium*.

All available type strains of the genus *Brachybacterium* deposited in the NCBI database as of 12 December 2025, together with strain DNPG3^T^, were included in the pan-genome analysis. The genomic G + C content across all examined *Brachybacterium* species was found to range from 67.28% to 73.10%. As additional genomes were incorporated, the total number of pan-gene clusters expanded to 32,821, whereas the number of core gene clusters declined to 261. The blue curve represents the pan-genome and demonstrates a continuous increase in the number of gene clusters with the sequential addition of genomes, indicating the ongoing discovery of novel gene families. The lack of a clear plateau suggests that the *Brachybacterium* pan-genome remains open, reflecting substantial genetic diversity and continuous gene acquisition among different lineages. In contrast, the red curve denotes the core genome and shows a rapid reduction during the initial stages of genome incorporation, followed by stabilization as additional genomes are included. This trend indicates the gradual exclusion of non-shared genes and suggests that *Brachybacterium* species possess a relatively small conserved core genome accompanied by a large and diverse accessory genome that contributes to ecological adaptability and evolutionary diversification (Fig. 6A).

The UpSet plot revealed pronounced heterogeneity in the distribution of strain-specific gene clusters among the analyzed *Brachybacterium* genomes, with the number of unique genes per strain ranging from as few as 11 to as many as 3405 (Fig. 6B). This broad variation reflects substantial genome plasticity within the genus and points to lineage-specific gene gain and loss events likely driven by ecological differentiation and niche adaptation.

For strain DNPG3^T^, a total of 2784 unique gene clusters were identified, represented by a prominent single-genome intersection (Fig. 6B). This substantial strain-specific gene repertoire indicates a large and distinct accessory genome that was absent from the other 30 type strains included in the analysis. The abundance of unique genes provides strong genomic evidence for the distinctiveness of DNPG3^T^. Such exclusive gene content is commonly associated with specialized metabolic capabilities, enhanced environmental adaptability, and potential occupation of a unique ecological niche. Moreover, limited exclusive gene sharing was observed between DNPG3^T^ and *Brachybacterium rhamnosum* strain JCM 11650^ T^, with only 25 shared gene clusters, highlighting the substantial divergence of their accessory genomes despite close evolutionary relatedness. Overall, the UpSet plot depicted a relatively conserved core genome accompanied by a highly variable accessory genome within *Brachybacterium*. The distinctive gene repertoire of DNPG3^T^ supported its assignment as a separate genomic lineage, potentially representing a novel species.

### Secondary metabolite

The biosynthetic gene clusters (BGCs) identified in the genome of strain DNPG3^T^ and its closely related species were summarized in Table 4 and Supplementary Table 4. A total of five BGCs were detected in the DNPG3^T^ genome, whereas the genomes of *B. zhongshanense* strain JB^T^, *B. atlanticum* DSM 28796^ T^, and *B. muris* strain C3H-21^ T^ contained two, four, and three BGCs, respectively. Among the BGC classes predicted by antiSMASH, the most prominent cluster in DNPG3^T^ was BGC2, which harbored genes associated with RRE-containing and azole-containing RiPPs. This was followed by BGC3, which comprises genes associated with thioamitide biosynthesis. BLASTp analysis indicated that the ectoine-related gene within BGC4 shared 88.15% sequence similarity to a characterized ectoine synthase (Supplementary Table 4). Ectoine is a well-established compatible solute that plays a crucial role in protecting cells from osmotic stress, desiccation, elevated salinity, and temperature extremes. The presence of this biosynthetic cluster suggests that strain DNPG3^T^ possesses adaptive mechanisms that enhance cellular stability and survival under osmotic and other abiotic stress conditions, consistent with previous reports^41^. Two terpene-precursor genes showing more than 78% sequence similarity to polyprenyl synthetase proteins were identified in the genome of strain DNPG3^T^ (Supplementary Table 4). Comparative genomic analysis across five *Brachybacterium* genomes revealed that terpene-precursor biosynthetic elements were conserved among all examined strains. In bacteria, terpenes are known to function as antimicrobial compounds and signaling molecules, thereby contributing to ecological fitness and competitive interactions within complex microbial communities.

**Table 4:** Comparative analysis of BGCs identified in the genome of strain DNPG3^T^ and those of selected closely related taxa.

Strain	Total BGCs	Secondary metabolite genes														
		**bottromycin**	**NI-siderophore**	**RiPP-like**	**resorcinol**	**NRPS**	**terpene-precursor**	**terpene**	**NAPPA**	**ectoine**	**T1PKS**	**T2PKS**	**T3PKS**	**azole-containing-RiPP**	**thioamitides**	**RRE-containing**
*Brachybacterium* *netajii *strain DNPG3^T^	5						1	1	1	1				1	1	1
*B. Zhongshanense* strain JB^T^	2			1			1									
B. *atlanticum* strain DSM28796^T^	4		1				1	1	1	1						
B. *muris* strain C3H-21^ T^	3	1					1	1		1						
B. *squillarum* strain M-6-3^ T^	7				1	1	1	2			1	1	1			

Taken together, the in silico prediction of biosynthetic gene clusters provides a genomic foundation for hypothesizing about the adaptive strategies of strain DNPG3^T^. Moreover, the composition and diversity of these clusters suggest the ecological fitness of DNPG3^T^ and highlighted its potential as a source of novel bioactive compounds with pharmaceutical and industrial applications.

## Conclusion

The Gram-positive, halotolerant strain DNPG3^T^ (= MTCC13125^T^) was isolated from contaminated water collected from the River Ganges at Hooghly, India. The strain demonstrated the ability to degrade PNP. Whole-genome analysis revealed the presence of multiple genomic islands, carbohydrate-active enzymes and biosynthetic gene clusters, indicating a capacity for secondary metabolite production. These genomic features highlight the biotechnological potential of the strain, reflecting its metabolic versatility and potential for industrial enzyme applications.

During comprehensive phenotypic and chemotaxonomic characterization, the strain exhibited a distinctive cellular fatty acid composition, notably the predominance of C_11:0_ and C_10:0_ 2-OH fatty acids, along with marked differences from the closely related *Brachybacterium zhongshanense* strain JB^T^. Based on detailed molecular phylogenetic analyses in conjunction with genomic, chemotaxonomic, and phenotypic evidence, strain DNPG3^T^ is proposed as represent a novel species of the genus *Brachybacterium*, for which the name *Brachybacterium netajii* sp. nov. is suggested.

### Description of *Brachybacterium netajii* sp. nov.

*Brachybacterium netajii* (ne.ta’ji.i N.L. gen. n. *netajii*, of Netaji, the Hindi/Bengali honorific title (meaning “respected leader”) by which Subhas Chandra Bose, Indian nationalist (1897–1945), is widely known. He strongly advocated technological self-reliance, modern education, industrial development, and scientific innovation as essential pillars for national liberation, security, and socio-economic advancement. His enduring legacy highlights the integration of scientific progress with the pursuit of freedom and nation-building). The species name *Brachybacterium netajii* has been registered with the SeqCode Registry (https://seqco.de/i:56045) and is included in the register list https://seqco.de/r:_zufbysb, which has been endorsed by expert curators of the SeqCode^42^.

The strain DNPG3^T^ represents a novel species within the genus *Brachybacterium*. On the basis of its distinct phenotypic traits, comprehensive phylogenetic analyses, whole-genome characterization, major menaquinone profile, and FAME analysis, strain DNPG3^T^ is proposed as a novel species, for which the name *Brachybacterium netajii* sp. nov. is introduced.

Cells are Gram-positive and non-motile. Colonies formed on PNP agar are yellowish-white and circular, whereas those on TSA are cream-colored. Growth occurs over a temperature range of 20–40 °C, with optimal growth observed at 30 °C. The strain tolerates up to 9% NaCl and grows well within a pH range of 5.8–8.0. The strain is oxidase-negative but catalase-positive and exhibits starch-hydrolyzing activity. Positive reactions was observed for β-glucosidase activity. The organism is capable of utilizing sucrose, D-xylose, D-glucose, D-mannose, L-arabinose, D-melibiose, D-maltose, D-trehalose, D-cellobiose, D-sorbitol, and D-mannitol as carbon sources. Positive reactions are recorded for adenosine assimilation, L-lactate utilization, and citrulline metabolism. In contrast, the strain tested negative for Ala-Phe-Pro arylamidase, glycine arylamidase, pyrrolidonyl arylamidase, proline arylamidase, *β-*alanine arylamidase, and Gly-Gly arylamidase. H_2_S production was not detected. Furthermore, the strain lacked enzymatic activities associated with *β*-N-acetylglucosaminidase, lipase, phosphatase, β-glucuronidase, *α*-galactosidase, *α*-glucosidase, *β*-galactosidase, and *β*-xylosidase. The strain failed to utilize D-tagatose, 5-keto-D-gluconate, succinate, palatinose, N-acetyl-D-glucosamine, L-malate, L-arabinitol, and L-lactate as substrates.

The cellular fatty acid profile is dominated by C_15:0_ anteiso (24.61%) as the most abundant, followed by C₁₁:₀ (21.06%), C_16:0_ iso (11.89%), C₁₆:₀ (11.58%), and C_17:0_ anteiso (11.24%). The predominant respiratory quinone is menaquinone MK-7, consistent with members of the genus *Brachybacterium*. The type strain, designated DNPG3^T^ (= MTCC 13125^ T^), was isolated from the River Ganges, W.B, India.

## Supplementary Information

## Data Availability

The bacterial strain was deposited in the Microbial Type Culture Collection (MTCC), India, and assigned the accession number MTCC 13,125. The whole-genome shotgun sequencing data were deposited in the GenBank database under the accession number JBSYXB000000000. The associated project and sample metadata are available under BioProject accession PRJNA1380898, BioSample accession SAMN53927870, and Sequence Read Archive (SRA) accession SRR36475856.  *Brachybacterium netajii* sp. nov. has been included in the SeqCode register list https://seqco.de/r:_zufbysb.
