# Supplementary material for: Polyphasic taxonomic characterization of Brachybacterium netajii sp. nov., a metabolically versatile bacterium isolated from the river Ganges, India
Source: Sci Rep. 2026 Jun 16;16:27613. doi: 10.1038/s41598-026-56775-0 (PMC13538518; doi:10.1038/s41598-026-56775-0)
Supplement: Supplementary file 1 [file 41598_2026_56775_MOESM1_ESM.pdf]

Brachybacterium netajii sp. nov.: A halotolerant bacterium capable of p-Nitrophenol degradation, isolated from the Ganges river, West Bengal, India

Submitted by ALAM, SK AFTABUL

Abstract

Brachybacterium netajii strain DNPG3 was isolated in 2018 from the Ganges River in West Bengal, India. The Gram-positive, halotolerant, and heavy metal tolerant bacterium, demonstrated the capability to biodegrade *p*-nitrophenol (PNP) catabolically. Analysis of 16S rRNA gene sequence indicated that *Brachybacterium zhongshanense* strain JBT was the closest relative of DNPG3, sharing 97.08% sequence similarity. Genome-based ANI value calculated using the EzBioCloud server revealed that *B. zhongshanense* JCM 15471 was the closest genomic relative (85.49%). These values were further substantiated by digital DNA–DNA hybridization (dDDH) estimates calculated using the GGDC server. Comprehensive biochemical, physiological, chemotaxonomic, and phylogenetic analyses supported the assignment of strain DNPG3 to the genus *Brachybacterium* while clearly distinguishing it from all currently described species within the genus. Accordingly, strain DNPG3 was proposed to represent a novel species, for which the name *Brachybacterium netajii* sp. nov. is suggested. The type strain was DNPG3T (= MTCC13125T).

Species *Brachybacterium netajii*

Etymology

[ne.ta'ji.i] **N.L. gen. n.** *netajii*, of Netaji, the Hindi/Bengali honorific title (meaning "respected leader") by which Subhas Chandra Bose, Indian nationalist (1897–1945), is widely known

Nomenclatural type

NCBI Assembly: GCA_054114245.1 [Ts]

Reference Strain

Strain scl0041734: MTCC13125 = DNPG3

Description

Cells are Gram-positive and non-motile. Colonies formed on PNP agar are yellowish-white and circular. Growth occurs over a temperature range of 20–40°C, with optimal growth observed at 30 °C. The strain tolerates up to 9% NaCl and grows well within a pH range of 5.8–8.0. The strain is oxidase-negative but catalase-positive and exhibits starch-hydrolyzing activity. Positive reactions was observed for β-glucosidase activity. The organism is capable of utilizing sucrose, D-xylose, D-glucose, D-mannose, L-arabinose, D-melibiose, D-maltose, D-trehalose, D-cellobiose, D-sorbitol, and D-mannitol as carbon sources. Positive reactions are recorded for adenosine assimilation, L-lactate utilization, and citrulline metabolism. In contrast, the strain tested negative for Ala-Phe-Pro arylamidase, glycine arylamidase, pyrrolidonyl arylamidase, proline arylamidase, β-alanine arylamidase, and Gly-Gly arylamidase. H2S production was not detected. Furthermore, the strain lacked enzymatic activities associated with *β*-N-acetylglucosaminidase, lipase, phosphatase, β-glucuronidase, *α*-galactosidase, *α*-glucosidase, *β*-galactosidase, and *β*-xylosidase. The strain failed to utilize D-tagatose, 5-keto-D-gluconate, succinate, palatinose, N-acetyl-D-glucosamine, L-malate, L-arabinitol, and L-lactate as substrates.

The cellular fatty acid profile is dominated by C15:0 Anteiso (24.61%) as the most abundant, followed by $C_{11:0}$ (21.06%), iso-$C_{16:0}$ (11.89%), $C_{16:0}$ (11.58%), and anteiso-$C_{17:0}$ (11.24%). The predominant respiratory quinone is menaquinone MK-7, consistent with members of the genus *Brachybacterium*. The type strain, designated DNPG3T (= MTCC 13125T), was isolated from the River Ganges, W.B, India.

Classification

Bacteria » *Actinomycetota* » *Actinomycetes* » *Micrococcales* » *Dermabacteraceae* » *Brachybacterium* » *Brachybacterium netajii*

Registry URL

https://seqco.de/i:56045
