# Supplementary material for: Polyphasic taxonomic characterization of Brachybacterium netajii sp. nov., a metabolically versatile bacterium isolated from the river Ganges, India
Source: Sci Rep. 2026 Jun 16;16:27613. doi: 10.1038/s41598-026-56775-0 (PMC13538518; doi:10.1038/s41598-026-56775-0)
Supplement: Supplementary file 2 [file 41598_2026_56775_MOESM6_ESM.pdf]

Type Strain Genome Server

Query strain	Subject strain	dDDH (d0, in %)	C.I. (d0, in %)	dDDH (d4, in %)	C.I. (d4, in %)	dDDH (d6, in %)	C.I. (d6, in %)	G+C content difference (in %)
'DNPG3 FASTA'	Brachybacterium zhongshanense JCM 15471	46.6	[43.2 - 50.0]	27.1	[24.8 - 29.6]	40.5	[37.5 - 43.5]	1.81
'DNPG3 FASTA'	Brachybacterium horti JCM 31742	25.9	[22.6 - 29.6]	22.6	[20.3 - 25.1]	24.2	[21.3 - 27.3]	0.31
'DNPG3 FASTA'	Brachybacterium atlanticum PhyBa CO2 2	23.7	[20.4 - 27.4]	22.6	[20.3 - 25.0]	22.5	[19.7 - 25.6]	0.27
'DNPG3 FASTA'	Brachybacterium rhamnosum JCM 11650	26.3	[22.9 - 29.9]	22.6	[20.3 - 25.0]	24.4	[21.6 - 27.5]	0.61
'DNPG3 FASTA'	Brachybacterium conglomeratum 5-2	24.4	[21.1 - 28.1]	22.5	[20.2 - 24.9]	23.0	[20.2 - 26.1]	0.28
'DNPG3 FASTA'	Brachybacterium paraconglomeratum KCTC 9916	24.4	[21.1 - 28.0]	22.4	[20.1 - 24.8]	23.0	[20.1 - 26.1]	0.21
'DNPG3 FASTA'	Brachybacterium alimentarium JCM 11607	20.4	[17.2 - 24.0]	22.4	[20.1 - 24.9]	19.8	[17.1 - 22.8]	2.15
'DNPG3 FASTA'	Brachybacterium squillarum M-6-3	24.1	[20.8 - 27.7]	22.3	[20.0 - 24.8]	22.7	[19.9 - 25.8]	0.34
'DNPG3 FASTA'	Brachybacterium sacelli DSM 14566	20.3	[17.1 - 23.9]	22.2	[19.9 - 24.6]	19.7	[17.0 - 22.7]	2.17
'DNPG3 FASTA'	Brachybacterium fresconis DSM 14564	20.6	[17.4 - 24.2]	22.1	[19.9 - 24.6]	19.9	[17.2 - 23.0]	1.92
'DNPG3 FASTA'	Brachybacterium faecium DSM 4810	24.0	[20.7 - 27.6]	22.0	[19.7 - 24.4]	22.6	[19.8 - 25.7]	0.39
'DNPG3 FASTA'	Brachybacterium halotolerans subsp. kimchii CBA3105T	19.2	[16.1 - 22.8]	21.8	[19.6 - 24.3]	18.8	[16.1 - 21.8]	0.66
'DNPG3 FASTA'	Brachybacterium saurashtrense DSM23186	23.4	[20.1 - 27.1]	21.7	[19.4 - 24.1]	22.1	[19.3 - 25.2]	0.32
