# Supplementary material for: Polyphasic taxonomic characterization of Brachybacterium netajii sp. nov., a metabolically versatile bacterium isolated from the river Ganges, India
Source: Sci Rep. 2026 Jun 16;16:27613. doi: 10.1038/s41598-026-56775-0 (PMC13538518; doi:10.1038/s41598-026-56775-0)
Supplement: Supplementary file 3 [file 41598_2026_56775_MOESM7_ESM.docx]

Supplementary Table 3

<https://bcb.unl.edu/dbCAN2_obsolete/data/blast/20260101100618/overview.txt>

**CAZymes analysis**

| **Gene ID** | **EC#** | **HMMER** | **dbCAN_sub** | **DIAMOND** | **Signalp** | **#ofTools** |
| --- | --- | --- | --- | --- | --- | --- |
| MGY5763591.1 | - | GT39(423-691) | GT39_e19 | GT39 | N | 3 |
| MGY5763592.1 | 2.4.1.25 | GH77(179-691) | GH77_e2 | GH77 | N | 3 |
| MGY5763675.1 | - | GT4(202-344) | GT4_e582 | GT4 | N | 3 |
| MGY5763692.1 | - | GH13_21(221-544) | GH13_e177 | GH13_21 | N | 3 |
| MGY5763693.1 | 3.2.1.20 | GH13_30(52-414) | GH13_e234 | GH13_30 | N | 3 |
| MGY5763697.1 | - | GT4(223-373) | GT4_e3987 | GT4 | N | 3 |
| MGY5763699.1 | - | GT4(196-334) | GT4_e40 | GT4 | N | 3 |
| MGY5763716.1 | - | GT4(230-381) | GT4_e3466 | GT4 | N | 3 |
| MGY5763717.1 | - | CE14(40-192) | CE14_e16 | N | N | 2 |
| MGY5763743.1 | 2.4.1.1 | GT35(236-662) | GT35_e8 | GT35 | N | 3 |
| MGY5763806.1 | - | GT28(191-345) | GT28_e141 | GT28 | N | 3 |
| MGY5763868.1 | - | GH15(131-289) | GH15_e54 | GH15 | N | 3 |
| MGY5763952.1 | 2.4.1.64 | GH65(348-726) | GH65_e2 | GH65 | N | 3 |
| MGY5764060.1 | - | GH3(143-368) | GH3_e102 | GH3 | Y(1-28) | 3 |
| MGY5764088.1 | 3.2.1.23 | GH2(47-556) | GH2_e112 | GH2 | N | 3 |
| MGY5764107.1 | 5.4.99.16 | GH13_16(44-398) | GH13_e0 | GH13_16 | N | 3 |
| MGY5764111.1 | 3.2.1.26 | GH32(16-328) | GH32_e102 | N | N | 2 |
| MGY5764122.1 | 3.2.1.22 | GH36(10-721) | GH36_e2 | GH36 | N | 3 |
| MGY5764161.1 | - | GH127(74-597) | GH127_e8 | GH127 | Y(1-25) | 3 |
| MGY5764198.1 | 3.2.1.41\|3.2.1.1\|3.2.1.68 | GH13_13(201-550)+GH13_13(1339-1736) | GH13_e183+CBM48_e30+GH13_e183 | CBM48+GH13+GH13_13 | Y(1-27) | 3 |
| MGY5764272.1 | - | GH85(152-485) | GH85_e2 | GH85 | N | 3 |
| MGY5764273.1 | - | GH3(116-357) | GH3_e229 | GH3 | Y(1-24) | 3 |
| MGY5764274.1 | 3.2.1.25 | GH5_18(29-308) | GH5_e224 | GH5_18 | N | 3 |
| MGY5764278.1 | 3.2.1.24 | GH38(270-522) | GH38_e38 | GH38 | N | 3 |
| MGY5764290.1 | 3.2.1.55 | GH43_27(340-622) | GH43_e116 | GH43_27 | N | 3 |
| MGY5764298.1 | - | GH42(20-397) | GH42_e2 | GH42 | N | 3 |
| MGY5764300.1 | 1.1.3.- | AA7(38-468) | AA7_e0 | N | N | 2 |
| MGY5764318.1 | - | GT4(204-342) | GT4_e3495 | N | N | 2 |
| MGY5764327.1 | 3.2.1.141\|3.2.1.1 | GH13_10(132-447) | CBM48_e0+GH13_e92 | CBM48+GH13_10 | N | 3 |
| MGY5764328.1 | 5.4.99.15 | GH13_26(31-313) | GH13_e105 | GH13_26 | N | 3 |
| MGY5764329.1 | 3.2.1.68\|2.4.1.25\|3.2.1.- | CBM48(10-99)+GH13_11(176-529) | CBM48_e3+GH13_e48 | CBM48+GH13_11 | N | 3 |
| MGY5764509.1 | - | GH109(11-192) | GH109_e5 | N | N | 2 |
| MGY5764522.1 | - | GH25(57-239) | GH25_e89 | N | N | 2 |
| MGY5764537.1 | - | GT5(73-390) | GT4_e1800 | GT4 | N | 3 |
| MGY5764548.1 | - | GT2(18-183) | GT2 | GT2 | N | 3 |
| MGY5764600.1 | 3.2.1.78\|3.2.1.4 | GH5_2(61-293)+CBM59(371-512) | GH5_e323+CBM59_e2 | CBM59+GH5_2 | Y(1-37) | 3 |
| MGY5764634.1 | - | GH42(16-381) | GH42_e32 | GH42 | N | 3 |
| MGY5764686.1 | - | CE14(23-125) | CE14_e39 | N | N | 2 |
| MGY5764693.1 | - | GH114(74-237) | GH114_e3 | N | Y(1-29) | 2 |
| MGY5764701.1 | - | GH42(34-403) | GH42_e32 | GH42 | N | 3 |
| MGY5764723.1 | N | AA3(127-676) | N | N | N | 1 |
| MGY5764760.1 | 3.2.1.1\|3.2.1.116\|- | GH13_32(81-344)+CBM25(509-581)+CBM20(627-707) | GH13_e240+CBM25_e4+CBM20_e38 | CBM20+CBM25+GH13_32 | Y(1-42) | 3 |
| MGY5764884.1 | 3.2.1.68\|2.4.1.25\|3.2.1.- | GH13_11(190-575) | CBM48_e3+GH13_e48 | CBM48+GH13_11 | N | 3 |
| MGY5764885.1 | - | GH13_3(222-440) | GH13_e41 | GH13_3 | N | 3 |
| MGY5764886.1 | 2.4.1.18 | CBM48(130-217)+GH13_9(277-577) | CBM48_e2+GH13_e200 | CBM48+GH13_9 | N | 3 |
| MGY5764891.1 | - | CE9(33-414) | CE9_e100 | CE9 | N | 3 |
| MGY5764908.1 | N | GH109(5-160) | N | N | N | 1 |
| MGY5764914.1 | 2.4.1.15 | GT20(11-474) | GT20_e1 | GT20 | N | 3 |
| MGY5765043.1 | - | GH4(81-206) | GH4_e15 | GH4 | N | 3 |
| MGY5765055.1 | - | GH4(11-187) | GH4_e21 | GH4 | N | 3 |
| MGY5765064.1 | 3.2.1.25 | GH2(31-786) | GH2_e24 | GH2 | N | 3 |
| MGY5765082.1 | 3.2.1.23 | GH2(99-711) | GH2_e127 | GH2 | N | 3 |
| MGY5765090.1 | - | GH109(2-153) | GH109_e2 | N | N | 2 |
| MGY5765094.1 | - | GH109(29-182) | GH109_e7 | N | N | 2 |
| MGY5765099.1 | 3.2.1.37 | GH3(121-343) | GH3_e50 | GH3 | N | 3 |
| MGY5765107.1 | 3.2.1.8\|3.2.1.136 | GH30_8(290-535) | GH30_e78 | CBM35+GH30_8 | N | 3 |
| MGY5765108.1 | 3.2.1.139\|3.2.1.131 | GH67(103-683) | GH67_e1 | GH67 | N | 3 |
| MGY5765109.1 | - | CE7(3-330) | CE7_e16 | CE7 | N | 3 |
| MGY5765115.1 | - | CE7(3-322) | CE7_e32 | CE7 | N | 3 |
| MGY5765116.1 | 3.2.1.177 | GH31(244-672) | GH31_e31 | GH31 | N | 3 |
| MGY5765117.1 | 3.2.1.120\|3.2.1.74 | GH3(46-279) | GH3_e179 | GH3 | N | 3 |
| MGY5765131.1 | 3.2.1.55 | GH51(51-553) | GH51_e19 | GH51 | Y(1-32) | 3 |
| MGY5765138.1 | 3.2.1.21 | GH1(5-388) | GH1_e38 | GH1 | N | 3 |
| MGY5765156.1 | N | CE1(172-409) | N | N | N | 1 |
| MGY5765177.1 | - | GH25(101-272) | GH25_e211 | N | N | 2 |
| MGY5765184.1 | 3.2.1.78 | GH5_8(52-304)+CBM59(371-512) | GH5_e22+CBM59_e0 | CBM10+GH5_8 | Y(1-39) | 3 |
| MGY5765218.1 | 3.2.1.151\|3.2.1.150 | GH74(147-258) | GH74_e1+GH74_e0+GH74_e0 | CBM5+GH74 | N | 3 |
| MGY5765238.1 | - | GH109(3-165) | GH109_e7 | N | N | 2 |
| MGY5765241.1 | 3.2.1.185 | GH127(28-621) | GH127_e0 | GH127 | N | 3 |
| MGY5765255.1 | 4.2.2.23\|4.2.2.24 | PL11(75-681) | PL11_e0 | CE12+PL11_1 | Y(1-54) | 3 |
| MGY5765256.1 | 4.2.2.24 | PL26(6-913) | PL26_e0 | PL26 | N | 3 |
| MGY5765257.1 | - | CE12(4-204) | CE12_e66 | N | N | 2 |
| MGY5765264.1 | - | GH35(18-320) | GH35_e48 | GH35 | N | 3 |
| MGY5765274.1 | - | GH27(155-396) | GH27_e15 | GH27 | N | 3 |
| MGY5765284.1 | 3.2.1.99 | GH43_4(98-447) | GH43_e38 | GH43_4 | Y(1-54) | 3 |
| MGY5765295.1 | 3.2.1.55 | GH51(16-518) | GH51_e0 | GH51 | N | 3 |
| MGY5765296.1 | 3.2.1.55 | GH51(30-526) | GH51_e19 | GH51 | N | 3 |
| MGY5765299.1 | - | GH43_10(6-277) | GH43_e224 | GH43_10 | N | 3 |
| MGY5765303.1 | 3.2.1.67 | GH28(47-383) | GH28_e10 | GH28 | N | 3 |
| MGY5765306.1 | 4.2.2.2 | PL1_6(238-451) | PL1_e88 | PL1_6 | Y(1-62) | 3 |
| MGY5765307.1 | N | CE19(142-350) | N | N | N | 1 |
| MGY5765327.1 | - | GH43_24(135-437) | GH43_e144 | GH43_24 | N | 3 |
| MGY5765328.1 | 3.2.1.-\|3.2.1.63 | GH95(3-776) | GH95_e1 | GH95 | N | 3 |
| MGY5765340.1 | 3.2.1.- | GH42(10-377) | GH42_e0 | GH42 | N | 3 |
| MGY5765341.1 | 3.2.1.21\|3.2.1.-\|3.2.1.37\|3.2.1.55\|3.2.1.23\|3.2.1.45 | GH3(38-258) | GH3_e197 | GH3 | N | 3 |
| MGY5765342.1 | 3.2.1.55\|- | GH43_12(9-294)+CBM91(327-497) | GH43_e73+CBM91_e27 | GH43_12 | N | 3 |
| MGY5765376.1 | - | GT2(194-321)+GT2(426-543) | GT2+GT2 | GT2 | N | 3 |
| MGY5765391.1 | - | GT2(13-140) | GT2 | N | N | 2 |
| MGY5765404.1 | - | GT4(558-715) | GT4_e722 | GT4 | N | 3 |
| MGY5765407.1 | 3.2.1.55\|- | GH43_12(25-304) | GH43_e73+CBM91_e27 | GH43_12 | N | 3 |
| MGY5765410.1 | - | GH79(125-414) | GH79_e24 | GH79 | Y(1-36) | 3 |
| MGY5765482.1 | - | GT4(197-331) | GT4_e2272 | GT4 | N | 3 |
| MGY5765506.1 | 3.2.1.23 | GH2(59-912) | GH2_e61 | GH2 | N | 3 |
| MGY5765516.1 | - | GT2(28-160) | GT2 | GT2 | N | 3 |
| MGY5765524.1 | - | GT2(152-399) | GT2 | GT2 | N | 3 |
| MGY5765531.1 | 3.2.1.25 | GH2(19-681) | GH2_e24 | GH2 | N | 3 |
| MGY5765533.1 | 3.2.1.78 | GH26(60-370)+CBM23(527-689) | GH26_e73+CBM23_e4 | CBM23+GH26 | Y(1-28) | 3 |
| MGY5765539.1 | 3.2.1.52 | GH20(87-438) | GH20_e51 | GH20 | N | 3 |
| MGY5765542.1 | - | GT51(84-272) | GT51_e127 | GT51 | N | 3 |
| MGY5765600.1 | - | GH109(5-196) | GH109_e7 | N | N | 2 |
| MGY5765611.1 | 3.2.1.52 | GH3(125-343) | GH3_e164 | N | N | 2 |
| MGY5765685.1 | - | GT4(197-307) | GT4_e791 | GT4 | N | 3 |
| MGY5765688.1 | - | GT4(171-313) | GT4_e1978 | GT4 | N | 3 |
| MGY5765689.1 | - | GT2(9-222)+GT4(541-668) | GT2+GT4_e1572 | GT4 | N | 3 |
| MGY5765691.1 | - | GT2(8-138) | GT2 | N | N | 2 |
| MGY5765694.1 | - | GT2(17-123) | GT2 | N | N | 2 |
| MGY5765751.1 | - | CE8(25-280)+CE12(323-526) | CE8_e94+CE12_e50 | CE12+CE8 | N | 3 |
| MGY5765765.1 | 3.2.1.8 | GH10(77-398) | GH10_e50 | GH10 | Y(1-27) | 3 |
| MGY5765770.1 | 3.2.1.-\|3.2.1.21 | GH3(565-780) | GH3_e14 | GH3 | N | 3 |
| MGY5765796.1 | 3.2.1.99 | GH43_5(99-375) | GH43_e109 | GH43_5 | N | 3 |
| MGY5765817.1 | 3.2.1.3 | GH15(321-690) | GH15_e59 | GH15 | N | 3 |
| MGY5765823.1 | 3.2.1.89\|- | GH53(48-405)+CBM61(412-553) | GH53_e15+CBM61_e9 | CBM61+GH53 | Y(1-42) | 3 |
| MGY5765824.1 | - | GH53(7-382) | GH53_e17 | GH53 | N | 3 |
| MGY5765828.1 | 3.2.1.23 | GH42(19-389) | GH42_e34 | GH42 | N | 3 |
| MGY5765829.1 | 3.2.1.23\|3.2.1.-\|3.2.1.38 | GH35(13-323) | GH35_e23 | GH35 | N | 3 |
| MGY5765912.1 | - | GT4(206-346) | GT4_e3764 | N | N | 2 |
| MGY5765915.1 | - | GT4(201-345) | GT4_e638 | GT4 | N | 3 |
| MGY5765924.1 | - | GT2(23-134) | GT2 | GT2 | N | 3 |
| MGY5765927.1 | - | GT2(43-172) | GT2 | GT2 | N | 3 |
| MGY5765987.1 | - | GH3(140-393) | GH3_e66 | GH3 | N | 3 |
| MGY5765989.1 | N | GH109(13-187) | N | N | N | 1 |
| MGY5766054.1 | - | CBM67(214-330)+GH78(421-925) | GH78_e84 | GH78 | N | 3 |
| MGY5766086.1 | - | GT51(205-375) | GT51_e150 | GT51 | N | 3 |
| MGY5766185.1 | - | CE4(368-476) | CE4_e303 | N | N | 2 |
| MGY5766311.1 | - | AA3_2(24-340) | AA3_e4 | AA3_2 | N | 3 |
| MGY5766328.1 | N | AA1(572-767) | N | N | N | 1 |
| MGY5766335.1 | - | GH5_28(12-211) | GH5_e308 | GH5 | N | 3 |
| MGY5766348.1 | 3.2.1.113 | GH92(406-914) | GH92_e1+CBM32_e90 | GH92 | N | 3 |
| MGY5766428.1 | - | GH125(38-491) | GH125_e2 | GH125 | N | 3 |
| MGY5766440.1 | - | GH43_26(29-335) | GH43_e175 | GH43_26 | N | 3 |
| MGY5766474.1 | 3.2.1.63\|3.2.1.51 | GH95(33-831) | GH95_e0 | GH95 | Y(1-35) | 3 |
| MGY5766497.1 | - | GH32(17-357) | GH32_e68 | GH32 | N | 3 |
| MGY5766509.1 | - | GH13_20(67-369) | GH13_e155 | GH13 | N | 3 |
| MGY5766510.1 | 2.3.1.122\|3.1.1.-\|2.3.1.20 | CE1(62-273) | CE1_e32 | N | N | 2 |
| MGY5766522.1 | 3.2.1.146 | GH43_30(69-333) | GH43_e265 | GH43_30 | Y(1-34) | 3 |
| MGY5766552.1 | 3.2.1.40\|3.1.1.73 | CBM67(151-314)+GH78(377-893) | CBM67_e28+GH78_e80 | CBM67+GH78 | N | 3 |
| MGY5766568.1 | - | GH31(184-607) | GH31_e77 | GH31 | N | 3 |
| MGY5766646.1 | 3.5.1.115 | CE14(15-150) | CE14_e1 | CE14 | N | 3 |
| MGY5766682.1 | - | GH109(2-151) | GH109_e7 | N | N | 2 |
| MGY5764193.1 | - | N | GT2 | GT2 | N | 2 |
| MGY5765393.1 | - | N | GT4_e3368 | N | N | 1 |
| MGY5765395.1 | - | N | GT2 | N | N | 1 |
| MGY5765680.1 | - | N | GT2 | GT2 | N | 2 |
| MGY5765692.1 | - | N | GT4_e4024 | N | N | 1 |
| MGY5765734.1 | - | N | GT2 | GT2 | N | 2 |
| MGY5765413.1 | - | N | GT2 | GT0 | N | 2 |
| MGY5765714.1 | - | N | GH23_e158 | N | Y(1-53) | 1 |
| MGY5765928.1 | - | N | GT2 | GT2 | N | 2 |
| MGY5765935.1 | - | N | GT2 | GT2 | N | 2 |
| MGY5765956.1 | - | N | CBM50_e215 | N | N | 1 |
| MGY5766402.1 | - | N | GT4_e732 | GT4 | N | 2 |
| MGY5766406.1 | - | N | GT4_e3432 | GT4 | N | 2 |
| MGY5765400.1 | - | N | GT4_e3080+GT4_e3368 | GT4 | N | 2 |
| MGY5765917.1 | - | N | GT4_e2723 | GT4 | N | 2 |
| MGY5765919.1 | - | N | GT4_e2973 | GT4 | N | 2 |
| MGY5766205.1 | - | N | GH23_e158 | GH23 | Y(1-27) | 2 |
| MGY5766336.1 | - | N | GH23_e744 | N | Y(1-47) | 1 |
| MGY5763575.1 | N | N | N | GH13_31 | N | 1 |
| MGY5763615.1 | N | N | N | GT4 | N | 1 |
| MGY5763653.1 | N | N | N | GH1 | N | 1 |
| MGY5763661.1 | N | N | N | GT4 | N | 1 |
| MGY5763664.1 | N | N | N | GH6 | N | 1 |
| MGY5763795.1 | N | N | N | GH13_30 | N | 1 |
| MGY5763843.1 | N | N | N | GT2 | N | 1 |
| MGY5763861.1 | N | N | N | GT2 | N | 1 |
| MGY5763866.1 | N | N | N | CBM50+GH25 | N | 1 |
| MGY5763971.1 | N | N | N | GT4 | N | 1 |
| MGY5764035.1 | N | N | N | GH13 | N | 1 |
| MGY5764102.1 | N | N | N | GH13 | N | 1 |
| MGY5764110.1 | N | N | N | GH13_31 | N | 1 |
| MGY5764135.1 | N | N | N | CBM48+GH13_9 | N | 1 |
| MGY5764142.1 | N | N | N | CE12 | N | 1 |
| MGY5764173.1 | N | N | N | CE9 | N | 1 |
| MGY5764331.1 | N | N | N | GH28 | N | 1 |
| MGY5764348.1 | N | N | N | GT83 | N | 1 |
| MGY5764618.1 | N | N | N | CBM50 | N | 1 |
| MGY5764666.1 | N | N | N | GH28 | N | 1 |
| MGY5764894.1 | N | N | N | GT58 | N | 1 |
| MGY5764922.1 | N | N | N | GT2 | N | 1 |
| MGY5765180.1 | N | N | N | CBM9 | N | 1 |
| MGY5765403.1 | N | N | N | PL0 | N | 1 |
| MGY5765434.1 | N | N | N | CBM5 | Y(1-41) | 1 |
| MGY5765678.1 | N | N | N | GT2 | N | 1 |
| MGY5765687.1 | N | N | N | GT4 | N | 1 |
| MGY5765768.1 | N | N | N | GH65 | N | 1 |
| MGY5765873.1 | N | N | N | GH13_11 | N | 1 |
| MGY5765902.1 | N | N | N | GH101 | N | 1 |
| MGY5766032.1 | N | N | N | CBM50 | N | 1 |
| MGY5766202.1 | N | N | N | GH1 | N | 1 |
| MGY5766208.1 | N | N | N | GH38 | N | 1 |
| MGY5766281.1 | N | N | N | GH43_29 | N | 1 |
| MGY5766398.1 | N | N | N | GT2 | N | 1 |
| MGY5766506.1 | N | N | N | CBM48+GH13_11 | N | 1 |
| MGY5766518.1 | N | N | N | PL0 | N | 1 |

glycoside hydrolases (GHs), glycosyltransferases (GTs), carbohydrate-binding molecules (CBMs), carbohydrate esterases (CEs), and auxiliary activities (AAs)
