# Supplementary material for: Polyphasic taxonomic characterization of Brachybacterium netajii sp. nov., a metabolically versatile bacterium isolated from the river Ganges, India
Source: Sci Rep. 2026 Jun 16;16:27613. doi: 10.1038/s41598-026-56775-0 (PMC13538518; doi:10.1038/s41598-026-56775-0)
Supplement: Supplementary file 4 [file 41598_2026_56775_MOESM8_ESM.docx]

Supplementary Table..

| **Strain Name** | **Cluster ID** | **Cluster Type** | **Genomic Location (Start–End)** | **Length (nt)** | **BLASTp identity** | **BLASTp**  **Product** |
| --- | --- | --- | --- | --- | --- | --- |
| *Brachybacterium* sp Strain DNPG3 | BGC1 | terpene-precursor | 81,494 - 82,651 | 1158 | 78.18% | Polyprenyl synthetase protein ([BGZ93848.1](https://www.ncbi.nlm.nih.gov/protein/BGZ93848.1?report=genbank&log$=prottop&blast_rank=1&RID=N31CHTHR016)) |
|  | BGC2 | RRE-containing | 380,416 - 382,371, | 1956 | 44.87% | Hypothetical Protein ([WP_142043591.1](https://www.ncbi.nlm.nih.gov/protein/WP_142043591.1?report=genbank&log$=prottop&blast_rank=1&RID=N31R92K8016)) |
|  |  | azole-containing-RiPP | 383802-391,409; 397,318 - 398,517 | 7607; 1200 | 65.08% | TOMM precursor leader peptide-binding protein ([WP_158645486.1](https://www.ncbi.nlm.nih.gov/protein/WP_158645486.1?report=genbank&log$=prottop&blast_rank=1&RID=N31TW197016)) |
|  | BGC3 | thioamitides | 440669-447,194 | 6525 | 61.69% | Yca-O family Protein ([WP_191727267.1](https://www.ncbi.nlm.nih.gov/protein/WP_191727267.1?report=genbank&log$=prottop&blast_rank=1&RID=N31WZEJN016)) |
|  | BGC4 | NAPAA | 357,290 - 361,243 | 3954 | 83.36% | Pls/PosA family peptide synthase  ([WP_137770042.1](https://www.ncbi.nlm.nih.gov/protein/WP_137770042.1?report=genbank&log$=prottop&blast_rank=1&RID=N321ZABH016)) |
|  |  | ectoine | 368,632 - 369,039 | 408 | 88.15% | Ectoine synthase ([WP_106267693.1](https://www.ncbi.nlm.nih.gov/protein/WP_106267693.1?report=genbank&log$=prottop&blast_rank=1&RID=N325RR2D016)) |
|  | BGC5 | terpene | 149,971-151,077; 153,436-154,347; 155,969-156,669; | 1107; 912; 700 | 98.08% | Polyprenyl synthetase family protein ([WP_329749341.1](https://www.ncbi.nlm.nih.gov/protein/WP_329749341.1?report=genbank&log$=prottop&blast_rank=1&RID=N328C5DH016)) |
|  |  |  |  |  |  |  |
| *B. Zhongshanense* strain JB^T^ | BGC1 | RiPP-like | 1,333 - 2,427, | 1095 | 99.12% | Hypothetical Protein ([BGZ93007.1](https://www.ncbi.nlm.nih.gov/protein/BGZ93007.1?report=genbank&log$=prottop&blast_rank=1&RID=N32VP5B1014)) |
|  | BGC2 | terpene-precursor | 343 - 741 | 399 | 96.58% | Polyprenyl synthetase family protein ([BGZ93027.](https://www.ncbi.nlm.nih.gov/protein/BGZ93027.1?report=genbank&log$=prottop&blast_rank=1&RID=N32XJ5TA014)1) |
|  |  |  |  |  |  |  |
| *B. atlanticum* strain_DSM28796^T^ | BGC1 | NAPAA, | 65,596 - 69,561; | 3966, | 96.53% | Pls/PosA family peptide synthase ([WP_440138710.1](https://www.ncbi.nlm.nih.gov/protein/WP_440138710.1?report=genbank&log$=prottop&blast_rank=1&RID=N32FTMU7016)) |
|  |  | ectoine | 62,572 - 62,964 | 393 | 96.92% | Ectoine synthase ([WP_396501093.1](https://www.ncbi.nlm.nih.gov/protein/WP_396501093.1?report=genbank&log$=prottop&blast_rank=1&RID=N32DD5ZN014)) |
|  | BGC2 | terpene-precursor | 209,129 - 210,262 | 1134 | 94.43% | Polyprenyl synthetase family protein ([WP_053917238.1](https://www.ncbi.nlm.nih.gov/protein/WP_053917238.1?report=genbank&log$=prottop&blast_rank=1&RID=N32JT4GV014)) |
|  | BGC3 | terpene | 2,691-3,863;4,926-5,804; 7,450-8,102 | 1173, 879, 657 | 93.59% | Polyprenyl synthetase family ([WP_062946412.1](https://www.ncbi.nlm.nih.gov/protein/WP_062946412.1?report=genbank&log$=prottop&blast_rank=1&RID=N32N4GBP014)) |
|  | BGC4 | NI-siderophore | 69,953 - 70,828 | 876 | 100% | LucA/LucC family protein ([WP_308445905.1](https://www.ncbi.nlm.nih.gov/protein/WP_308445905.1?report=genbank&log$=prottop&blast_rank=1&RID=N32RAXH5014)) |
|  |  |  |  |  |  |  |
| *B. muris* strain C3H-21^T^ | BGC1 | ectoine | 10,303-10,680 | 387 | 91.20% | Ectoine synthase  ([WP_412020692.1](https://www.ncbi.nlm.nih.gov/protein/WP_412020692.1?report=genbank&log$=prottop&blast_rank=1&RID=N3324U3A016)) |
|  |  | bottromycin | 14,577-15,380 | 804 | 94.38% | Alpha/beta fold hydrolase  ([WP_259804142.1](https://www.ncbi.nlm.nih.gov/protein/WP_259804142.1?report=genbank&log$=prottop&blast_rank=1&RID=N3354TZ2016)) |
|  | BGC2 | terpene | 1516-2,195; 3,826-5,849 | 679; 2023 | 98.05% | Phytoene/squalene synthase family protein ( [WP_259841745.1](https://www.ncbi.nlm.nih.gov/protein/WP_259841745.1?report=genbank&log$=prottop&blast_rank=1&RID=N3387W30016)) |
|  | BGC3 | terpene-precursor | 17,494 - 18,669, | 1176 | 99.49% | Polyprenyl synthetase family protein ([WP_017823668.1](https://www.ncbi.nlm.nih.gov/protein/WP_017823668.1?report=genbank&log$=prottop&blast_rank=1&RID=N33B79H4014)) |
|  |  |  |  |  |  |  |
| *B. squillarum* strain M-6-3^T^ | BGC1 | terpene | 340,430-341,130 | 700 | 100.00% | Lycopene cyclase family Protein ([WP_010534716.1](https://www.ncbi.nlm.nih.gov/protein/WP_010534716.1?report=genbank&log$=prottop&blast_rank=1&RID=N33G400T016)) |
|  | BGC2 | terpene-precursor | 387,483 - 388,616, | 1134 | 100.00% | Polyprenyl synthetase family protein ([WP_010534400.1](https://www.ncbi.nlm.nih.gov/protein/WP_010534400.1?report=genbank&log$=prottop&blast_rank=1&RID=N33JV7JM016)) |
|  | BGC3 | T3PKS | 501,560 - 502,783 | 1224 | 100.00% | [hydroxymethylglutaryl-CoA synthase](https://blast.ncbi.nlm.nih.gov/Blast.cgi#alnHdr_WP_010532958) ([WP_010532958.1](https://www.ncbi.nlm.nih.gov/protein/WP_010532958.1?report=genbank&log$=prottop&blast_rank=1&RID=N33MKUVS016)) |
|  | BGC4 | resorcinol | 547,367 - 549,022, | 1656 | 100.00% | Redox enzyme gfamily Protein (  [WP_010532993.1](https://www.ncbi.nlm.nih.gov/protein/WP_010532993.1?report=genbank&log$=prottop&blast_rank=1&RID=N33PTF34016)) |
|  | BGC5 | terpene | 988874-990,924 | 2050 | 100.00% | Phytoene/squalene synthase family protein ([WP_010533371.1](https://www.ncbi.nlm.nih.gov/protein/WP_010533371.1?report=genbank&log$=prottop&blast_rank=1&RID=N33TYMAY014)) |
|  | BGC6 | NRPS, T1PKS | 1,122,302 - 1,138,330, | 16029 | 100.00% | Type 1 polyketide synthase ([WP_280990798.1](https://www.ncbi.nlm.nih.gov/protein/WP_280990798.1?report=genbank&log$=prottop&blast_rank=1&RID=N33W0NBJ016)) |
|  | BGC7 | T2PKS | 1319782-1,322,024, | 2242 | 100% | beta-ketoacyl synthase Protein ([WP_010533660.1](https://www.ncbi.nlm.nih.gov/protein/WP_010533660.1?report=genbank&log$=protalign&blast_rank=1&RID=N33YWMS9014)**)** |
